# Multicomponent nursing interventions and frailty-related outcomes in older adults: An integrative review of primary studies and evidence syntheses

**DOI:** 10.1016/j.ijnsa.2026.100687

**Published:** 2026-10-01

**Authors:** Dhika Dharmansyah, Laili Rahayuwati, Iqbal Pramukti, Kuswandewi Mutyara, Sehabudin Salasa

**Affiliations:** a Doctoral Study Program in Medical Science, Faculty of Medicine, Universitas Padjadjaran, Jl. Raya Bandung-Sumedang Km. 21, Sumedang, West Java, Indonesia; b Department of Nursing, Faculty of Sport and Health Education, Universitas Pendidikan Indonesia, Jl. Dr. Setiabudhi No 229, Bandung, West Java, Indonesia; c Department of Community Health Nursing, Faculty of Nursing, Universitas Padjadjaran, Jl. Raya Bandung-Sumedang Km. 21, Sumedang, West Java, Indonesia; d Department of Public Health, Faculty of Medicine, Universitas Padjadjaran, Jl. Raya Bandung-Sumedang Km. 21, Sumedang, West Java, Indonesia

**Keywords:** Frailty, Nurses, Multicomponent intervention, Older adults, Integrative review, Comprehensive geriatric assessment

## Abstract

**Background:**

Frailty is a prevalent, dynamic and potentially modifiable syndrome in older adults, and nurses are increasingly involved in leading, delivering and coordinating the multicomponent interventions studied for this population. However, the typology of these interventions, the changes in frailty-related outcomes they report, the specific contributions of nurses, and the representation of low- and middle-income country evidence have not been integrated across diverse study designs.

**Objective:**

To integrate quantitative, qualitative and review-level evidence on nurse-led, nurse-delivered or nurse-coordinated multicomponent interventions for frailty in adults aged 60 years or older, and to characterise the nurse's role and the reported changes in frailty-related outcomes.

**Design:**

Integrative review following the Whittemore and Knafl (2005) framework, reported in accordance with the Preferred Reporting Items for Systematic Reviews and Meta-Analyses (PRISMA) 2020 statement and prospectively registered (Open Science Framework).

**Methods:**

Scopus and PubMed were searched on 15 January 2026 (316 records). In response to peer review, the search strategy was redesigned to remove overly restrictive requirements, broaden frailty terminology and separate the exercise and nutrition concepts; the revised PubMed strategy was re-run on September 2026 (13,663 records, screened in full), and verification searches were executed in EBSCOhost and SpringerLink. Backward citation searching of retained evidence syntheses was performed. All previously included studies were re-adjudicated at the full-text level against revised operational criteria requiring a documented frailty, pre-frailty or validated frailty-risk criterion and a defined minimum level of nursing involvement. Full texts were obtained for all retained primary studies. Methodological quality was appraised with the Mixed Methods Appraisal Tool (2018) using design-specific criteria, and evidence syntheses were appraised separately. The 23 retained primary studies constitute the principal analytic corpus; the 12 evidence syntheses are reported in a separate contextual section.

**Results:**

Of 316 records identified in the original search (273 screened after duplicate removal), 32 reports previously judged eligible were re-adjudicated. The updated PubMed search identified 13,663 records; after automated removal of 252 ineligible publication types, 13,411 records were screened, 44 reports were sought for retrieval, 23 were not retrieved and 21 were assessed at full text, of which 12 were excluded and 9 met all eligibility criteria. Thirty-five studies were included: 23 primary studies (9 randomised trials, 7 quasi-experimental, 3 mixed-methods, 4 observational) and 12 evidence syntheses. Six reports were excluded at re-adjudication: four lacked a documented frailty or pre-frailty criterion and two could not be obtained as full texts. Across the primary corpus, one trial reported a significant between-group categorical transition from pre-frailty toward robust status, three reported significant between-group improvements in validated continuous frailty scores, two quasi-experimental studies reported significant between-group improvements in non-validated or composite frailty scores, one pilot trial found no significant between-group effect, and four studies reported within-group improvements without between-group testing. Ten studies did not assess frailty status as an outcome. Five themes were generated: (1) a typology of multicomponent nursing interventions; (2) frailty outcomes and reported changes in frailty status; (3) the nurse's role and competencies; (4) implementation barriers and facilitators; and (5) geographic distribution of the evidence.

**Conclusions:**

Nurse-led, nurse-delivered and nurse-coordinated multicomponent interventions show promising associations with improvements in some frailty-related outcomes in controlled studies, most consistently for pre-frail community-dwelling older adults. The available evidence does not yet establish that nursing interventions reverse frailty trajectories, and effect estimates have not been pooled. Evidence from low- and middle-income countries remains very limited.

**What is already known**

• Frailty in older adults is a dynamic syndrome that may be prevented or partially modified.
• Multicomponent interventions, often built around comprehensive geriatric assessment, are recommended over single-domain approaches.
• Nurses are frequently involved in delivering and coordinating these interventions.

**What this paper adds**

• It re-adjudicates and re-characterises the primary evidence base using full texts and explicit operational criteria for frailty status and nursing involvement, separating 23 primary studies from 12 contextual evidence syntheses.
• It classifies reported frailty-related outcomes by evidential strength, distinguishing categorical transitions in frailty status from continuous score changes, domain-limited improvements and the absence of between-group effects.
• It documents the marked underrepresentation of low- and middle-income country evidence and quantifies the overlap between the primary corpus and retained evidence syntheses in a citation matrix.

## 1 Introduction

Frailty is a clinically recognisable state of increased vulnerability that results from age-associated declines in physiological reserve and function across multiple body systems, leaving older adults less able to cope with acute or everyday stressors (Clegg et al., 2013; Hoogendijk et al., 2019). Two dominant operationalisations, the physical frailty phenotype (Fried et al., 2001) and accumulation-of-deficits frailty index models, underpin most screening and intervention work. Frailty is understood not as a fixed endpoint but as a dynamic continuum along which individuals may move toward greater robustness or further decline, which provides the conceptual rationale for intervention (Hoogendijk et al., 2019; Dent et al., 2019).

The global burden of frailty is substantial and growing. Prevalence estimates among community-dwelling older adults vary widely with setting and definition, with the highest estimates reported from low- and middle-income countries (LMICs) (Siriwardhana et al., 2018). The incidence of frailty among non-frail community-dwelling older adults is estimated at 43.4 new cases per 1000 person-years, with higher rates in women and in LMICs (Ofori-Asenso et al., 2019). In a prospective analysis of 493,737 UK Biobank participants, frailty and pre-frailty were associated with multimorbidity and with increased all-cause mortality (Hanlon et al., 2018). By 2050, approximately 21.3% of the global population will be 60 years or older (United Nations, 2019), amplifying the public health imperative for effective prevention and management strategies.

Contemporary frailty scholarship recognises that the syndrome extends beyond physical decline. A multidimensional frailty model encompasses physical (sarcopenia, functional limitation, gait slowing), cognitive (mild cognitive impairment, dementia risk), psychological (depression, anxiety, low self-efficacy) and social domains (isolation, social vulnerability), as well as clinical complexity factors such as multimorbidity and polypharmacy (Clegg et al., 2013; Hoogendijk et al., 2019). Multimorbidity and frailty commonly co-occur and jointly worsen outcomes including mortality, nursing home admission and need for home care (Carrasco-Ribelles et al., 2022), and polypharmacy is prospectively associated with higher frailty risk (Veronese et al., 2017). Falls and fear of falling further compound functional decline; fear of falling is common among community-dwelling older adults and is associated with frailty (Xiong et al., 2024; de Souza et al., 2022). These interconnected domains underscore the limitations of single-component interventions and strengthen the rationale for multicomponent approaches that involve nursing.

This multidimensional understanding aligns with the World Health Organization's (WHO) Integrated Care for Older People (ICOPE) framework, which positions the maintenance and optimisation of intrinsic capacity, encompassing locomotion, cognition, psychological well-being, vitality and sensory function, as the central strategy for healthy ageing (World Health Organization, 2019). Frailty and intrinsic capacity decline share overlapping determinants, and the ICOPE framework explicitly prioritises multicomponent, person-centred care pathways in which nurses are well placed to participate (Cesari et al., 2018; WHO, 2019).

International guidance increasingly recommends multicomponent interventions, typically combining structured physical activity with nutritional, educational, psychosocial or medication-optimisation elements, over single-domain approaches for managing physical frailty (Dent et al., 2019). Nurses participate in these models in several distinct capacities: through comprehensive geriatric assessment (CGA), care coordination, health coaching and the direct delivery of exercise and lifestyle components, they are among the most consistently involved professionals in frailty care pathways (Fulceri et al., 2026; Sum et al., 2022). Nurses' longitudinal relationships with patients, their capacity to contribute to holistic assessment across physical, cognitive, psychological and social domains, and their presence across primary care, community, hospital and long-term care settings position them as potentially central contributors within multicomponent frailty programmes. The literature describing this contribution is, however, fragmented across heterogeneous designs and settings, and it is dispersed between high-income countries (HICs) and a comparatively sparse LMIC evidence base.

No single methodology fully captures this diversity. An integrative review is appropriate because it permits the simultaneous synthesis of experimental, observational, qualitative and review-level evidence to develop a comprehensive understanding of a complex phenomenon (Whittemore and Knafl, 2005). In line with peer review of an earlier version of this manuscript, this review treats primary studies as the principal analytic corpus and reports evidence syntheses separately, and it interprets outcome evidence using between-group estimates rather than statistical significance alone.

The primary objective of this review was to characterise the typology of nurse-led, nurse-delivered or nurse-coordinated multicomponent interventions for frailty in adults aged 60 years or older, and to describe the reported changes in frailty-related outcomes associated with these interventions. The secondary objectives were to: (a) delineate the specific roles and competencies that nurses contribute, using an explicit taxonomy of nurse-led, nurse-delivered and nurse-coordinated models; (b) identify the implementation barriers and facilitators associated with these programmes; and (c) map the geographic distribution of the evidence, with particular attention to the representation of LMIC settings.

The review addressed four questions: (1) What is the typology of nurse-involved multicomponent frailty interventions? (2) What changes in frailty status or frailty-related outcomes are reported in controlled studies, and with what evidential strength? (3) What roles and competencies do nurses contribute? (4) What are the implementation barriers and facilitators, and how well are LMIC and other underrepresented contexts represented?

## 2 Methods

### 2.1 Design and eligibility criteria

This integrative review followed the Whittemore and Knafl (2005) framework and is reported in accordance with the PRISMA 2020 statement (Page et al., 2021). The protocol was registered prospectively on the Open Science Framework on 10 December 2025 (DOI: 10.17605/OSF.IO/B4RCJ); deviations from the protocol are documented in Section 2.7.

Studies were eligible if they examined a multicomponent intervention, defined as at least two intervention components including at least one physical activity or exercise component, delivered wholly or in part by nurses, in which the intervention targeted frailty in adults with a mean age of 60 years or older, or with a minimum eligible age of 60 years. Eligible designs were randomised trials, quasi-experimental studies, observational studies, mixed-methods studies and evidence syntheses of such studies. Reviews were retained as contextual sources, reported separately from the primary corpus (Section 3.8); meta-analyses were not eligible for outcome synthesis, consistent with the integrative design.

Two operational criteria were applied, in revised form, at re-adjudication. First, frailty criterion: studies were required to document frailty, pre-frailty or elevated frailty risk at baseline using a validated instrument (for example the Fried physical frailty phenotype, the FRAIL scale, the Tilburg Frailty Indicator, a frailty index, the Korean Frailty Index or an established geriatric assessment composite) or an explicit operational definition or validated risk-prediction model reported in the full text. The previously used, less specific category of being 'at risk of frailty' without a documented criterion was removed. Second, nursing involvement: eligible studies were required to document, in the full text, at least one of three nursing roles. Nurse-led refers to models in which nurses hold primary responsibility for the design, decision-making and delivery of the intervention. Nurse-delivered refers to models in which nurses deliver one or more defined intervention components. Nurse-coordinated refers to models in which nurses coordinate assessment, referral, follow-up or multidisciplinary team processes with delivery shared across professionals. Studies in which nurses could not be confirmed as involved at this level, or in which the frailty criterion was not documented, were excluded.

Publications in languages other than English were eligible if an English abstract or full text permitted verification of these criteria and the study characteristics. Publications from 2015 onward were eligible. Trial registrations, protocol publications and conference abstracts were used to verify study details but were not separately included.

### 2.2 Search strategy

The original search was executed in Scopus and PubMed on 15 January 2026 using two concept blocks (frailty terms; multicomponent intervention terms), with results restricted to 2015 onward; it retrieved 316 records (Scopus n = 270, PubMed n = 46). In response to peer review, the strategy was redesigned. The revised strategy (1) removed the requirement that records contain nursing-role terminology, because the nursing contribution is frequently documented only in the full text, and instead assesses the nursing criterion at full-text appraisal; (2) separated the previously co-occurring exercise and nutrition concept into independent terms combined with Boolean OR; (3) broadened the frailty free-text set to include pre-frail, pre-frailty and frailty syndrome variants in addition to Medical Subject Headings (MeSH) terms; and (4) added parallel strings for CINAHL and Embase. The complete revised strings for all databases are provided in Supplementary Table S5.

The revised PubMed strategy was re-run on September 2026 and retrieved 13,663 records, compared with 46 records retrieved by the original PubMed string, confirming that the original strategy was substantially under-sensitive. To validate the revised strategy, a corrected four-study known-item set was assembled from studies that meet all revised eligibility criteria and that were identified during the earlier stages of this review: Zheng et al. (2024, a systematic review of nurse-led interventions for pre-frail or frail older adults published in Ageing Research Reviews), Kasa et al. (2023), Song and Boo (2022) and Yas and Secginli (2025). All four known items were retrieved by the revised PubMed strategy (4 of 4). In the previous version of this manuscript, the known-item set had been described with an incorrect journal attribution for one item and a reference that did not resolve; these errors are corrected here. The revised strategy was additionally executed in EBSCOhost (8398 records, 2015 onward) and SpringerLink (40,614 records, 2015 onward) on 20 September 2026 to verify coverage of records not indexed in PubMed; no additional eligible primary study was apparent among the top-ranked results of either search, and both were used as verification searches rather than exhaustive screening sources. Parallel translated strings for Scopus, CINAHL and Embase are provided in Supplementary Table S5 for execution through institutional access. Backward citation searching was performed by examining the reference lists of all retained evidence syntheses for primary studies not captured by the database searches, and forward citation searching of the retained primary studies was performed. Duplicates across databases were removed using reference management software before screening.

### 2.3 Study selection

Records retrieved by the original search were screened at title and abstract level between 15 January and 12 February 2026; a 20% random sample was independently double-screened, with disagreements resolved through discussion and arbitration by a second reviewer. Because the per-record dual-screening decisions were not retained, chance-corrected agreement statistics cannot be computed and reported; the quality-assurance procedures and the agreed transparent reporting approach are documented in Supplementary Table S3. Full-text assessment of the 32 reports previously judged eligible was then re-adjudicated against the revised operational criteria between 1 and 10 September 2026 by two reviewers working independently, with disagreements resolved through discussion and arbitration. The term full-text screening is used consistently for this stage. The 13,663 records identified by the updated PubMed search were screened at title and abstract level following automated removal of 252 ineligible publication types; candidate reports were retrieved and assessed at full text against the same criteria, and 23 potentially eligible reports that could not be retrieved (paywalled despite retrieval attempts via repositories, author manuscripts and DOI resolution) are documented in Supplementary Table S4. The screening and selection process is documented in the PRISMA 2020 flow diagram (Fig. 1), in which exclusion reasons at the full-text re-adjudication stage are reported separately from title and abstract exclusions.

**Fig. 1:**
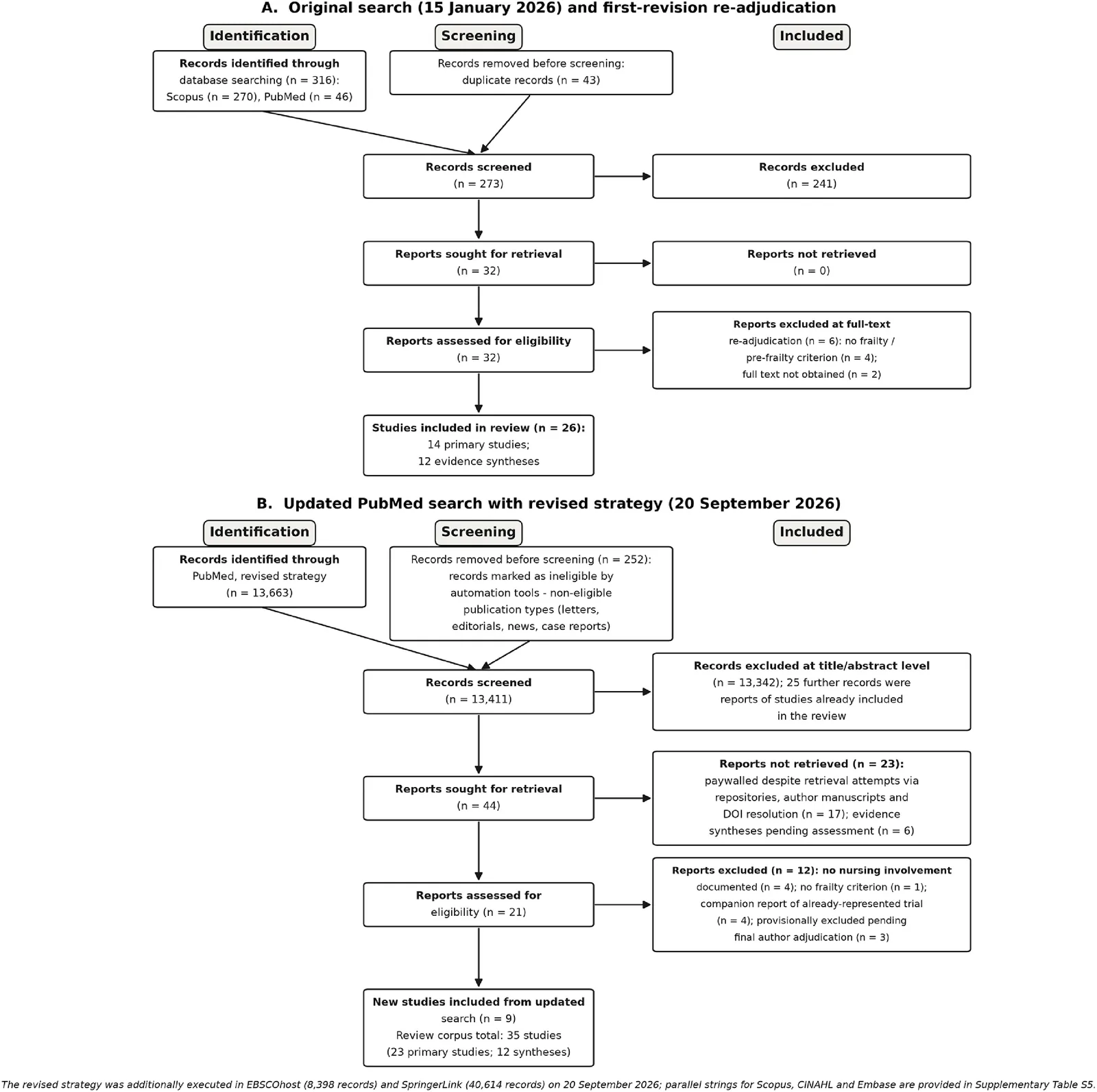
PRISMA 2020 flow diagram of study identification, screening, re-adjudication and inclusion (n = 35 included: 23 primary studies and 12 evidence syntheses).

### 2.4 Data extraction and full-text retrieval

A standardised form was used to extract study characteristics (design, country, setting, sample, frailty criterion, nursing role, components, duration, outcomes, results, registration) and review-level characteristics (aims, databases, quality appraisal approach, registration, included studies). All extractions were performed from full texts; where the full text reported no frailty eligibility criterion, this was coded as such. Full texts were obtained for all 14 retained primary studies: 10 through open-access publications or repositories and 4 (Bleijenberg 2016; Bleijenberg 2017; Ettema 2015 and Huang 2025) through institutional access, supplemented by verification against trial registrations (ClinicalTrials.gov NCT04332887 for Huang 2025) and companion publications of the same trials. Two reports could not be obtained as full texts (Hongyan et al., 2022; Liang et al., 2023); in line with peer review, these are documented rather than treated as fully analysed studies. A second reviewer verified a 20% random sample of extractions against source documents; discrepancies were resolved by re-reading the full text. Where study characteristics were verified through registration documents or companion publications in addition to the primary report, this is indicated in Supplementary Table S1.

### 2.5 Methodological quality appraisal

The 23 primary studies were appraised with the Mixed Methods Appraisal Tool (MMAT; Hong et al., 2018) using the design-specific criteria for each of the four MMAT categories represented in the corpus (randomised trials, non-randomised studies, quantitative descriptive studies and mixed-methods studies). In line with MMAT guidance, each design-specific criterion was rated (yes, no or can't tell) without computing overall scores, and the ratings are summarised narratively in Section 3.1 and reported in full in Supplementary Table S2. Where a criterion could not be assessed because it was not addressed in the source documents, it was rated can't tell rather than yes; this rating was used, for example, for allocation concealment in three trials. The 12 evidence syntheses were appraised separately with the AMSTAR 2 (Shea et al., 2017), reported in Section 3.8 and Supplementary Table S8. Consistent with the integrative design, methodological ratings were used to contextualise the synthesis rather than to exclude studies. No hierarchy-of-evidence claim is made: review-level sources are reported as contextual evidence, not as stronger evidence than the primary studies.

### 2.6 Data synthesis

Quantitative, qualitative and review-level data were integrated using constant comparison and a staged matrix synthesis (Whittemore and Knafl, 2005). Extracted data were coded by the first reviewer, and the coding of a 20% random sample was verified independently by a second reviewer; coding discrepancies were resolved through discussion. The synthesis is based on the 23 primary studies as the principal analytic corpus; the 12 evidence syntheses are reported separately in Section 3.8 to avoid conflating review-level and primary evidence. For studies reporting frailty outcomes, results were classified by evidential strength using an operational scheme: (a) categorical transitions in frailty status with between-group testing; (b) significant between-group improvement in a validated continuous frailty score; (c) improvement limited to a non-validated or composite frailty score; (d) absence of a significant between-group effect on the frailty outcome; (e) within-group improvement reported without between-group testing; (f) descriptive improvement reported without an inferential between-group test; and (g) frailty status not assessed as an outcome. Estimates are reported with 95% confidence intervals where available. No pooled effect estimates were computed. Counts reported across the manuscript were reconciled against the study table (Table 1) and the PRISMA flow (Fig. 1).

**Table 1:** Characteristics of the 23 included primary studies (verified at full-text level).

**Study (country)**	**Design**	**Population and frailty criterion**	**Nursing role**	**Intervention components**	**Frailty-related outcome**	**Reported result**
Faria et al. (2023), Portugal	Randomised trial	Community-dwelling older adults with frailty (Tilburg Frailty Indicator)	Nurse-led	Home-based advanced practice rehabilitation nursing programme: exercise, education, psychosocial support (12 weeks)	Tilburg Frailty Indicator total and domain scores	Within-group improvements; no between-group test reported
Yas and Secginli (2025), Turkiye	Randomised trial	Pre-frail women aged 55 years or older (FRAIL scale), n = 84	Nurse-led	Salutogenic programme: exercise, nutrition, education, psychosocial coaching (6 weeks)	FRAIL scale; frailty transitions	Significant between-group FRAIL improvement at 3, 6 and 9 months (p < 0.001); about half of the intervention group transitioned toward robust status
Kim et al. (2025), Republic of Korea	Pilot randomised trial	Adults aged 70 years or older with frailty (Korean Frailty Index for Primary Care at least 0.25), n = 84	Nurse-delivered	Integrated care programme: exercise, nutrition, social support, care coordination	Korean Frailty Index for Primary Care	Group-by-time interaction significant (p = 0.009); between-group frailty index change non-significant at 3 and 6 months
Luo et al. (2024), China	Randomised trial	Older stroke patients with frailty (FRAIL scale score of 3 or more), n = 125	Nurse-led	Multicomponent exercise nursing programme: progressive exercise, nutrition, education (12 weeks)	FRAIL scale	Significant between-group FRAIL differences at 4 and 12 weeks (both p < 0.001)
Huang et al. (2025), Taiwan	Randomised trial	Older adults after cardiac surgery, pre-frail or frail (Fried phenotype), n = 92	Nurse-delivered	Home-based multicomponent exercise with nutrition and nursing consultation (12 weeks)	Fried phenotype score	Significantly greater between-group frailty score improvement at 6 and 12 weeks
Zhou et al. (2025), China	Non-randomised emulated target trial with propensity-score matching	Older adults after percutaneous coronary intervention (Fried-based frailty phenotype), n = 136 matched	Nurse-led	Seven-component digital cardiac rehabilitation: exercise, education, nutrition, remote follow-up	Frailty phenotype distribution; six-minute walk, gait speed, grip strength	Descriptive phenotype improvement (pre-frail 100% to 75%); baseline frailty imbalance between groups; no inferential between-group frailty test
Song and Boo (2022), Republic of Korea	Quasi-experimental (non-equivalent control group)	Community-dwelling older adults living alone with frailty (frailty score threshold)	Nurse-led	Multicomponent programme: exercise, nutrition, education (12 weeks)	Composite frailty score	Significant group-by-time improvement (p = 0.009, partial eta-squared = 0.073); instrument not a validated frailty scale; no confidence intervals reported
Bleijenberg et al. (2017), Netherlands	Three-arm cluster randomised trial	Community-dwelling older adults screened with an electronic frailty index (39 practices, n = 3092)	Nurse-led	Frailty screening plus nurse-led proactive care programme based on comprehensive geriatric assessment	Daily functioning, quality-adjusted life-years, costs	No frailty-status outcome; favourable cost-effectiveness probability for the nurse-led arm
Bleijenberg et al. (2016), Netherlands	Mixed-methods fidelity study	Primary care older adults in the U-PROFIT programme	Nurse-coordinated	Nurse-led proactive programme components	Fidelity of component delivery	Variable delivery of components; nutrition counselling least often delivered
Muntinga et al. (2015), Netherlands	Fidelity and implementation study within a stepped-wedge cluster randomised trial	Frail community-dwelling older adults (composite geriatric screening)	Nurse-coordinated	Geriatric assessment and multidisciplinary care	Adherence to assessments and care plans	Assessment adherence initially high then declined; multidisciplinary consultation adherence improved over time
Ettema et al. (2015), Netherlands	Mixed-methods feasibility study	Older cardiac surgery candidates (prehabilitation)	Nurse-delivered	Prehabilitation: exercise, nutrition, education	Feasibility outcomes	Feasible; integration into routine cardiac care difficult
Azzellino et al. (2026a), Italy	Retrospective cohort	Frail older inpatients (Brass scale 11 or more), n = 200	Nurse-coordinated	Integrated discharge pathway: early home care activation plus nurse case management	30-day readmission	Nurse case manager involvement associated with lower readmission (odds ratio 0.023, 95% confidence interval 0.008 to 0.064, p < 0.001)
Ron et al. (2025), Israel	Prospective observational study with comparison group	Older adults with elevated frailty risk before surgery (validated screening)	Nurse-delivered	Comprehensive geriatric assessment plus prehabilitation delivered by a geriatric nurse	Activities of daily living and instrumental activities of daily living	Non-significant between-group differences (p = 0.13 to 0.15)
Cypel et al. (2026), Israel	Retrospective cohort	Adults aged 65 years or older at frailty risk (validated prediction model), n = 135,445	Nurse-coordinated	Comprehensive geriatric assessment plus nursing follow-up programme	Hip fractures, hospitalisation days, vaccination coverage	Before-after improvements (hip fractures down 46.77%); no concurrent control group
Jepma et al. (2021), Netherlands	Randomised controlled trial	Hospitalised older adults screened with the Discharge Screening for Community-dwelling frail older adults scale (2 points or more), n = 306	Nurse-coordinated	Care Chain Bailiwick: nurse-coordinated transitional care - comprehensive geriatric assessment, case management and home-based cardiac rehabilitation	Activities of daily living; mortality; readmission	Sustained activities of daily living benefit at 12 months; frailty not assessed as an outcome
Ha and Park (2020), Republic of Korea	Quasi-experimental (non-equivalent control group)	Community-dwelling pre-frail older adults (Cardiovascular Health Study criteria), n = 40	Nurse-led	Person-centred nursing intervention: exercise, nutrition and mental health education (8 weeks)	Cardiovascular Health Study frailty index; grip strength; Short Physical Performance Battery	Significant between-group improvements in frailty index, grip strength and physical function
Kasa et al. (2024), Ethiopia	Quasi-experimental (single group)	Community-dwelling older people with frailty, n = 66	Nurse-led	Six-session nurse-led programme spanning physical, nutritional, psychological and social domains	Frailty; depression; quality of life; activities of daily living	Significant within-group reduction in frailty and depression at 12 weeks
Kang et al. (2025), Republic of Korea	Quasi-experimental (single group, pretest-posttest)	Community-dwelling older adults, 100% pre-frail or frail (Elderly Health Interview Survey frailty instrument), n = 92	Nurse-led	Community health nurse-led multi-faceted group programme: education, exercise and social participation	Frailty (continuous and categorical); functional capacity	Significant within-group improvement in frailty scores and frailty categories
Lee et al. (2025), Republic of Korea	Pragmatic quasi-experimental trial	Community-dwelling pre-frail older adults (Korean frailty scale), n = 40	Nurse-led	Nursing Frailty Prevention programme: nurse counselling, group education, exercise and walking	Frailty score and domains	Significant between-group reduction in frailty scores, with improvements in physical and social domains
Safari et al. (2023), United Kingdom	Mixed-methods pilot randomised controlled trial	Community-dwelling older adults with frailty (electronic frailty index), n = 72	Nurse-led	Comprehensive geriatric assessment and care support plan delivered by advanced nurse practitioners in primary care	Feasibility and acceptability; health and care usage; functional outcomes	Feasible and acceptable; secondary outcomes did not change significantly; frailty not assessed as an outcome
Hayes et al. (2025), Ireland	Prospective cohort	Community-dwelling older adults with Clinical Frailty Scale scores 4–6, n = 303	Nurse-delivered	Community specialist teams: interdisciplinary comprehensive geriatric assessment including advanced nurse practitioners and a clinical nurse specialist	Functional status; mortality, hospitalisation and nursing home admission	Frailty predicted adverse outcomes; frailty not assessed as an outcome
van Lieshout et al. (2018), Netherlands	Randomised controlled trial	Community-dwelling pre-frail or frail older adults (Groningen Frailty Indicator 1 or more), n = 281 (139/142)	Nurse-delivered	SPRY programme: medication review, physical fitness training, social skills training (community nurse) and nutrition	Daily functioning (Katz-6); well-being	No statistically significant between-group difference in daily functioning at 12 months
Yoo et al. (2026), Republic of Korea	Single-group pretest-posttest pilot	Nursing home residents aged 65 years or older meeting Fried criteria (Korean Mini-Mental State Examination 18 or more), n = 27	Nurse-led	Nurse-led peer support programme with structured motor-cognitive activities (6 weeks)	Feasibility; handgrip strength; depressive symptoms; social frailty	Feasible and acceptable; within-group changes in social frailty and secondary outcomes

### 2.7 Protocol deviations

The following deviations from the registered protocol are reported for transparency. First, the original search string required nursing terminology in the same record as frailty and intervention terms, which rendered the strategy under-sensitive; the strategy was redesigned and re-run as described in Section 2.2. Second, the operational eligibility criteria were tightened at re-adjudication to require a documented frailty or pre-frailty criterion and a defined minimum level of nursing involvement, resulting in the exclusion of four previously included primary studies and the separate documentation of two reports whose full texts could not be obtained. Third, following peer review, the primary studies and evidence syntheses are now analysed in separate sections, and the outcome classification scheme and the appraisal approach (MMAT design-specific criteria for primary studies, AMSTAR 2 for syntheses) are reported in full. Fourth, the reported study counts were recalculated and reconciled across all tables and figures.

## 3 Results

### 3.1 Study selection

Of 316 records identified in the original search (Scopus n = 270; PubMed n = 46), 43 duplicates were removed and 273 records were screened at title and abstract level, of which 241 were excluded. Thirty-two reports previously judged eligible were re-adjudicated at the full-text level against the revised operational criteria (Section 2.1). Six reports were excluded at re-adjudication: four because no frailty, pre-frailty or validated frailty-risk criterion was documented (Alcolea-Ruiz et al., 2026, which recruited by fear-of-falling status; Orcel et al., 2025, which enrolled adults aged 70 years or older with chronic conditions; Irimia et al., 2022, a service audit in which frailty was assessed only post hoc; Esser et al., 2026, a heart failure population), and two because full texts could not be obtained despite contact with authors and retrieval attempts (Hongyan et al., 2022; Liang et al., 2023; documented in Supplementary Table S6). The updated PubMed search on 20 September 2026 retrieved 13,663 records and retrieved all four items in the corrected known-item validation set. After automated removal of 252 records marked as ineligible publication types, 13,411 records were screened at title and abstract level and 13,342 were excluded, of which 25 were reports of studies already included in the review. Forty-four reports were sought for retrieval; 23 could not be retrieved as full texts (documented in Supplementary Table S4) and 21 were assessed for eligibility, of which 12 were excluded (four with no documented nursing involvement, one with no frailty or pre-frailty eligibility criterion, four companion reports of already-represented trials and three provisionally excluded pending final adjudication; Supplementary Table S4). Nine additional studies met all eligibility criteria and were included. Thirty-five studies were therefore included: 23 primary studies (the principal analytic corpus) and 12 evidence syntheses (reported separately in Section 3.8). The full selection process is shown in Fig. 1.

### 3.2 Characteristics of the primary studies

The 23 primary studies were conducted in the Republic of Korea (n = 6), the Netherlands (n = 6), Portugal (n = 1), Türkiye (n = 1), China (n = 2), Taiwan (n = 1), Italy (n = 1), Israel (n = 2), the United Kingdom (n = 1), Ireland (n = 1) and Ethiopia (n = 1). By design, there were 9 randomised trials, 7 quasi-experimental studies, 3 mixed-methods studies and 4 observational studies (Fig. 2). Twenty studies were conducted in high-income countries, two in an upper-middle-income country (China) and one in a lower-middle-income country (Ethiopia). Settings included primary care and community programmes (n = 16), hospital or perioperative pathways (n = 5), home care (n = 1) and nursing homes (n = 1). Frailty was ascertained with the Fried phenotype or a derivative (n = 6), the FRAIL scale (n = 2), the Tilburg Frailty Indicator (n = 1) or the Korean Frailty Index for Primary Care (n = 1), with the remainder using validated screening or risk instruments (including the Groningen Frailty Indicator, the Clinical Frailty Scale, the Brass scale and discharge screening instruments) or study-specific operational definitions reported in the full text. Study characteristics are summarised in Table 1.

**Fig. 2:**
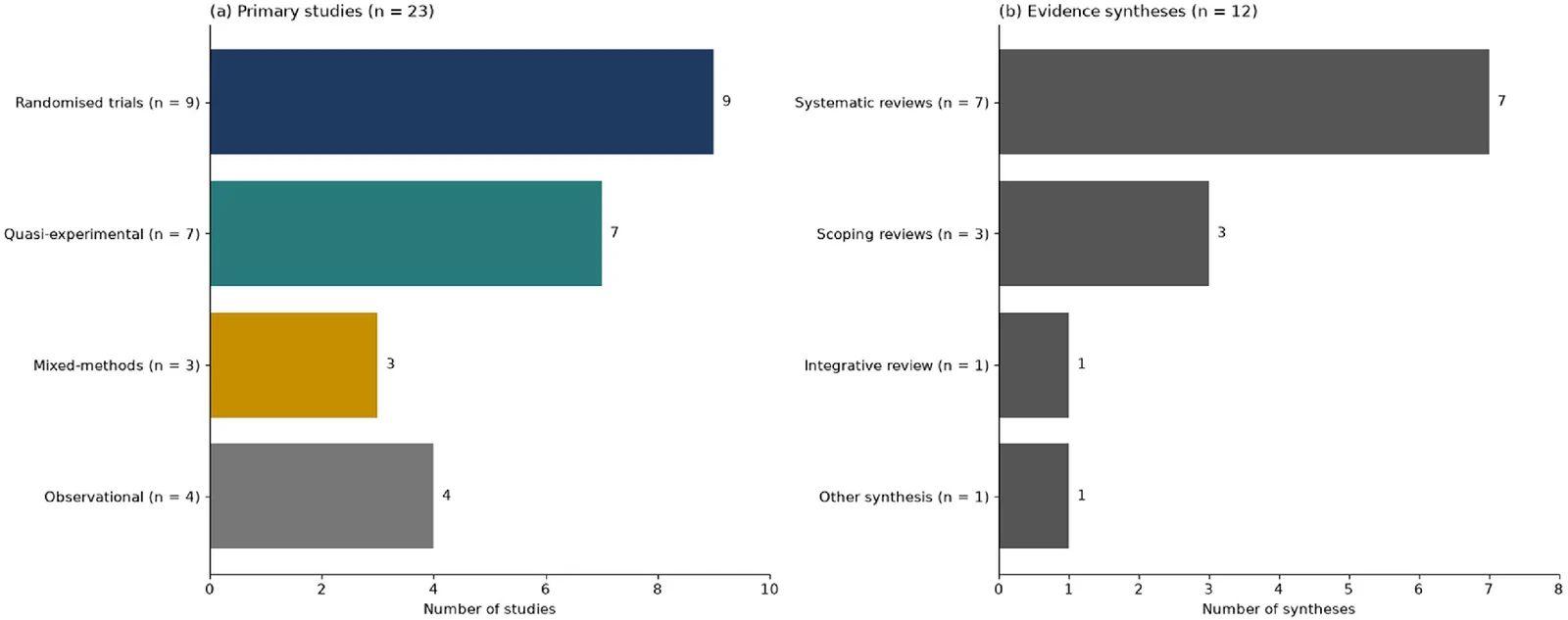
Study designs of the included primary studies (panel a) and evidence syntheses (panel b).

Methodological quality appraised with MMAT design-specific criteria is summarised narratively. Of the 9 randomised trials, all reported an appropriate randomisation process; allocation concealment could not be confirmed from the published reports of three trials (rated can't tell), and blinding of outcome assessors was documented in three trials. Completeness of outcome data was acceptable in all nine trials. Of the 7 quasi-experimental studies, two addressed confounding analytically (mixed-model adjustment in Song and Boo, 2022; propensity-score matching in Zhou et al., 2025), although neither achieved randomised comparability, and the groups in Zhou et al. (2025) were imbalanced at baseline on frailty status; the five quasi-experimental studies added in the updated search (Ha and Park, 2020; Kasa et al., 2024; Kang et al., 2025; Lee et al., 2025; Yoo et al., 2026) used non-equivalent control or single-group designs, three of which included no control group. Of the 3 mixed-methods studies, integration of quantitative and qualitative components was appropriate in all three. Of the 4 observational studies, confounding was addressed statistically in Azzellino et al. (2026a) but not in Cypel et al. (2026), whose before-after design lacked a concurrent control group; Ron et al. (2025) used a non-randomised comparison group and a small sample; and Hayes et al. (2025) was a single-arm prospective cohort without a concurrent control group. Full MMAT ratings are provided in Supplementary Table S2. No studies were excluded on the basis of methodological quality.

### 3.3 Theme 1: a typology of multicomponent nursing interventions

The 23 primary studies evaluated interventions comprising between two and seven components. The most common combination was structured exercise with nutrition and education (n = 7), followed by exercise with education alone (n = 5), comprehensive geriatric assessment-based care programmes (n = 7) and digitally delivered multicomponent programmes (n = 2), with the remaining programmes combining medication review, psychosocial support and social-participation components (n = 3). Exercise components ranged from supervised progressive resistance and balance training (Faria et al., 2023; Luo et al., 2024) to tailored home-based programmes of up to 12 weeks (Huang et al., 2025; Zhou et al., 2025). Nutritional components comprised screening plus counselling and, in some trials, oral nutritional supplementation (Yas and Secginli, 2025; Huang et al., 2025). Educational and psychosocial components included frailty education, goal setting, motivational support and salutogenic coaching (Yas and Secginli, 2025; Song and Boo, 2022). Programmes structured explicitly around comprehensive geriatric assessment included the nurse-led primary care studies added in the updated search (Safari et al., 2023; Hayes et al., 2025), with nurses conducting or coordinating assessment and follow-up (Muntinga et al., 2015; Ron et al., 2025; Cypel et al., 2026). Using the role taxonomy defined in Section 2.1, twelve programmes were nurse-led (Faria et al., 2023; Yas and Secginli, 2025; Song and Boo, 2022; Luo et al., 2024; Zhou et al., 2025; Bleijenberg et al., 2017; Ha and Park, 2020; Kasa et al., 2024; Kang et al., 2025; Lee et al., 2025; Yoo et al., 2026; Safari et al., 2023), six were nurse-delivered within multidisciplinary teams (Huang et al., 2025; Kim et al., 2025; Ettema et al., 2015; Ron et al., 2025; Hayes et al., 2025; van Lieshout et al., 2018) and five were nurse-coordinated (Azzellino et al., 2026a; Cypel et al., 2026; Muntinga et al., 2015; Bleijenberg et al., 2016; Jepma et al., 2021).

### 3.4 Theme 2: frailty outcomes and reported changes in frailty status

This theme was renamed from 'mechanisms of frailty reversal' to describe what the included studies actually report: changes in frailty status or frailty-related outcomes, with and without between-group testing. Applying the classification scheme defined in Section 2.6 (Table 2, Fig. 3), one randomised trial reported a categorical transition in frailty status with significant between-group testing: in Yas and Secginli (2025), 6 weeks of a nurse-led salutogenic programme for pre-frail women produced between-group FRAIL score improvements at 3, 6 and 9 months (t = −5.05, −4.71 and −4.09, all p < 0.001, eta-squared = 0.28), and approximately half of the intervention group transitioned toward robust status compared with none of the control group (p < 0.001). Two randomised trials reported significant between-group improvements in validated continuous frailty scores: Luo et al. (2024) found FRAIL scale differences at 4 and 12 weeks in stroke patients with frailty (t = 7.882 and 11.629, both p < 0.001; group-by-time F = 47.82, eta-squared = 0.388), and Huang et al. (2025) reported greater improvement in Fried phenotype scores at 6 and 12 weeks after multicomponent home exercise with nursing consultation following cardiac surgery. One quasi-experimental study reported a significant group-by-time improvement in a composite frailty score (Song and Boo, 2022; p = 0.009, partial eta-squared = 0.073), although the instrument was not a validated frailty scale and no confidence intervals were reported.

**Table 2:** Classification of reported frailty-related outcomes in the 23 primary studies.

**Category**	**Definition**	**Studies**	**n**
a	Categorical transition in frailty status with significant between-group testing	Yas and Secginli (2025)	1
b	Significant between-group improvement in a validated continuous frailty score	Luo et al. (2024); Huang et al. (2025); Ha and Park (2020)	3
c	Improvement in a non-validated or composite frailty score with significant between-group or group-by-time change	Song and Boo (2022); Lee et al. (2025)	2
d	No significant between-group effect on the frailty outcome	Kim et al. (2025)	1
e	Within-group improvement reported without between-group testing	Faria et al. (2023); Kasa et al. (2024); Kang et al. (2025); Yoo et al. (2026)	4
f	Descriptive improvement reported without an inferential between-group test	Zhou et al. (2025)	1
*g*	Frailty status not assessed as an outcome	Bleijenberg et al. (2016, 2017); Muntinga et al. (2015); Ettema et al. (2015); Ron et al. (2025); Azzellino et al. (2026a); Cypel et al. (2026); Jepma et al. (2021); van Lieshout et al. (2018); Safari et al. (2023); Hayes et al. (2025)	11

**Fig. 3:**
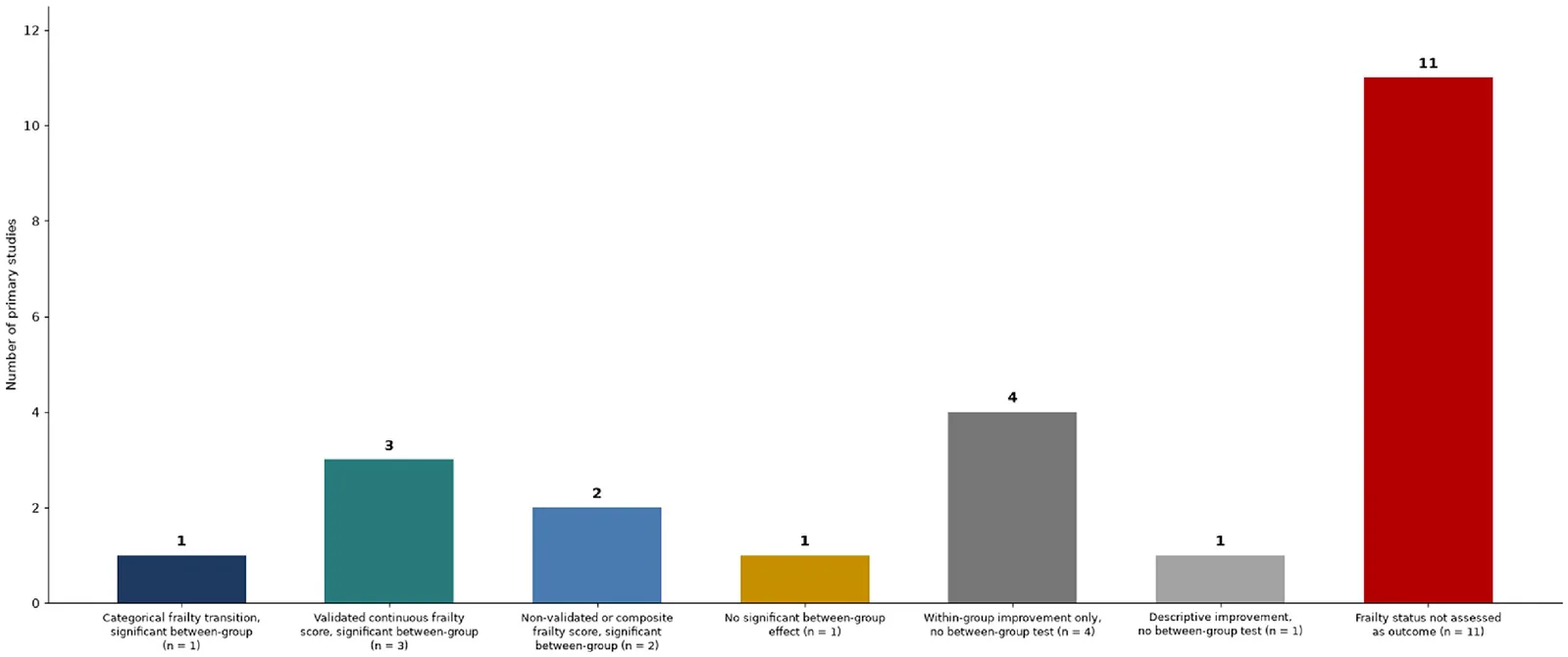
Classification of reported frailty-related outcomes in the 23 primary studies, by evidential strength of the reported change.

One pilot randomised trial found no significant between-group effect on the frailty outcome: in Kim et al. (2025), the between-group change in the Korean Frailty Index for Primary Care did not reach statistical significance at 3 months (Hedges' g = −0.42) or 6 months (g = −0.13), despite a significant group-by-time interaction in the overall model (F = 4.99, p = 0.009). One trial reported only within-group improvements in Tilburg Frailty Indicator domains without between-group testing (Faria et al., 2023). One non-randomised study reported descriptive improvement in frailty phenotype distribution without an inferential between-group test, with baseline imbalance in frailty status between groups (Zhou et al., 2025). Among the studies added in the updated search, one quasi-experimental study reported significant between-group improvement in the Cardiovascular Health Study frailty index, alongside grip strength and physical function (Ha and Park, 2020), and one pragmatic quasi-experimental trial reported a significant reduction in frailty scores compared with the control group (Lee et al., 2025). Three single-group studies reported significant within-group improvements in frailty scores without between-group testing (Kasa et al., 2024; Kang et al., 2025; Yoo et al., 2026), and one additional randomised trial found no statistically significant between-group difference in daily functioning at 12 months (van Lieshout et al., 2018). Ten studies did not assess frailty status as an outcome, reporting instead functional, service-use, implementation, feasibility or economic outcomes: these included the U-PROFIT programme's daily functioning, quality-adjusted life-year and cost outcomes (Bleijenberg et al., 2017), its fidelity analysis (Bleijenberg et al., 2016), the ACT programme's adherence outcomes (Muntinga et al., 2015), prehabilitation feasibility (Ettema et al., 2015), activities of daily living outcomes after nurse-led prehabilitation (Ron et al., 2025), readmission outcomes after nurse case-managed discharge (Azzellino et al., 2026a) and population-level service outcomes of a comprehensive geriatric assessment and nursing follow-up programme (Cypel et al., 2026). In Azzellino et al. (2026a), nurse case manager involvement was associated with lower 30-day readmission (odds ratio 0.023, 95% confidence interval 0.008 to 0.064, p < 0.001). Taken together, controlled evidence for improvement in frailty status is limited to a minority of trials, is concentrated in pre-frail community-dwelling populations, and does not support an inference of frailty reversal.

### 3.5 Theme 3: the nurse's role and competencies

Nurses functioned as assessors, coordinators, deliverers and providers of continuity. In comprehensive geriatric assessment-based pathways, nurses and advanced practice nurses conducted assessments, identified frailty and new medical issues, and led referral and discharge planning (Muntinga et al., 2015; Ron et al., 2025; Cypel et al., 2026; Safari et al., 2023). Nurse case management was associated with markedly lower 30-day readmission odds in one cohort (Azzellino et al., 2026a) and organised transitional care across hospital and home settings in another trial (Jepma et al., 2021). Twelve programmes placed nurses in lead roles with responsibility for design, decision-making and delivery (Section 3.3), including advanced practice or rehabilitation nurses delivering structured home or primary care programmes (Faria et al., 2023; Yas and Secginli, 2025; Safari et al., 2023). In six nurse-delivered models, nurses executed exercise, education and follow-up components within multidisciplinary teams (Huang et al., 2025; Kim et al., 2025; Ettema et al., 2015; Ron et al., 2025; Hayes et al., 2025; van Lieshout et al., 2018), and in five nurse-coordinated models they organised assessment, referral and continuity across professionals (Azzellino et al., 2026a; Cypel et al., 2026; Muntinga et al., 2015; Bleijenberg et al., 2016; Jepma et al., 2021).

### 3.6 Theme 4: implementation barriers and facilitators

Implementation determinants were prominent in the fidelity, feasibility and observational studies. Adherence to assessments and care plans was initially high but declined over 12 months, whereas adherence to multidisciplinary consultation improved over time (Muntinga et al., 2015). Treatment-fidelity analysis of the U-PROFIT programme revealed variable delivery of components, with nutrition counselling least often delivered (Bleijenberg et al., 2016). Prehabilitation was judged feasible, but integration into routine cardiac care pathways was difficult (Ettema et al., 2015). Economic analyses suggested favourable cost profiles for nurse-led and screening-plus-nurse models (Kim et al., 2025; Bleijenberg et al., 2017). Among the studies added in the updated search, the advanced nurse practitioner-delivered comprehensive geriatric assessment pilot was judged feasible and acceptable in United Kingdom primary care (Safari et al., 2023), the community health nurse-led group programme highlighted the contribution of two-nurse teams to successful delivery (Kang et al., 2025), and the nurse-led peer support pilot in nursing homes was feasible and acceptable (Yoo et al., 2026).

### 3.7 Theme 5: geographic distribution of the evidence

Twenty of the 23 primary studies were conducted in high-income countries, two in an upper-middle-income country (China) and one in a lower-middle-income country (Ethiopia) (Fig. 4); no low-income country evidence met the inclusion criteria. No eligible study was identified from South-East Asia. The updated search added the first African-region primary study to the corpus (Kasa et al., 2024, Ethiopia), and only limited additional African-region and Eastern Mediterranean-region evidence was identified in the retained syntheses, none of which met the inclusion criteria for the primary corpus. The evidence base therefore remains heavily concentrated in high-income, predominantly European and East Asian settings, and the generalisability of findings to low- and middle-income health systems is limited.

**Fig. 4:**
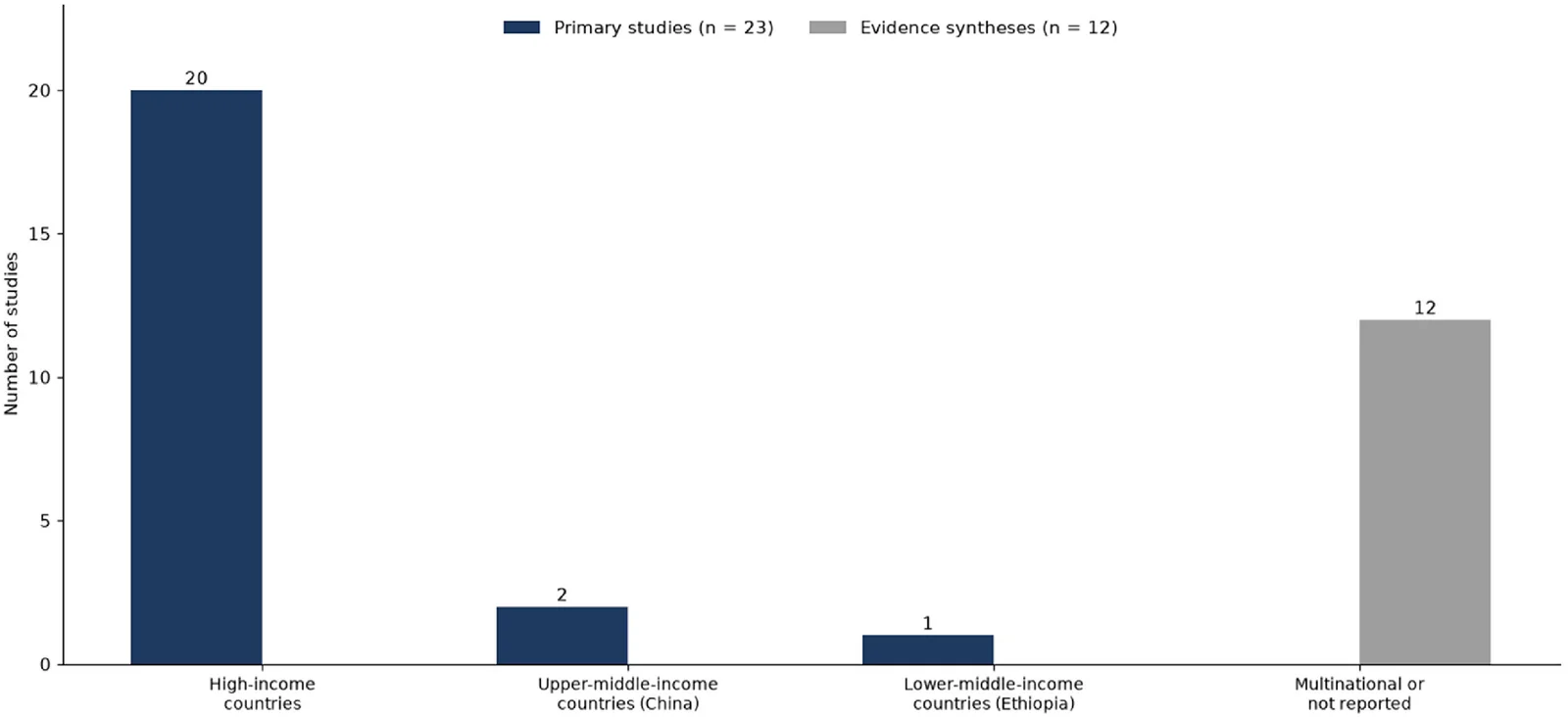
World Bank income classification of the settings of the included primary studies and evidence syntheses.

Table 4

### 3.8 Evidence syntheses: characteristics, appraisal and overlap with the primary corpus

The 12 retained evidence syntheses comprised 7 systematic reviews, 3 scoping reviews, 1 integrative review and 1 other synthesis (Table 3). Their scopes covered nurse-led interventions for frailty (Kasa et al., 2023), complex interventions and comprehensive geriatric assessment (Morkisch et al., 2020; Sum et al., 2022; Del Moral-Pairada et al., 2025), hospital implementation (Manuel et al., 2025), frailty interventions among community-dwelling older adults (Guan et al., 2025; Xu et al., 2024), nursing care models (Azzellino et al., 2026b) and regional and specialist contexts (Choi et al., 2023; Chen et al., 2026; Fulceri et al., 2026; Antunes et al., 2023). Appraised with AMSTAR 2 (Supplementary Table S8), seven syntheses reported prospective registration (six PROSPERO, one Open Science Framework); five did not report registration. Independent dual study selection was reported by six syntheses; one reported dual selection with single data extraction (Sum et al., 2022), and one reported no critical appraisal of included studies (Azzellino et al., 2026b). For four syntheses available to us only as paywalled full texts, appraisal relied on abstracts and registration records, and the corresponding ratings should be interpreted with caution. Citation searching across the syntheses' reference lists identified no additional primary study meeting all revised eligibility criteria. Overlap between the syntheses and the primary corpus was limited and is documented in a citation matrix (Supplementary Table S7): Del Moral-Pairada et al. (2025) and Sum et al. (2022) included reports from the U-PROFIT programme (Bleijenberg et al., 2016; 2017), and Kasa et al. (2023) included Song and Boo (2022); no other primary corpus study was included in any retained synthesis.

**Table 3:** Characteristics of the 12 retained evidence syntheses and their overlap with the primary corpus.

**Synthesis**	**Design**	**Focus**	**Registration**	**Selection and extraction**	**Appraisal of included studies**	**Overlap with primary corpus**
Kasa et al. (2023)	Systematic review	Nurse-led interventions for frailty in community-dwelling older people	PROSPERO CRD42022348064	Dual selection and dual extraction	Reported	Song and Boo (2022)
Morkisch et al. (2020)	Systematic review	Comprehensive geriatric assessment and complex interventions	PROSPERO CRD42018084604	Dual selection	Cochrane risk-of-bias tool	None
Sum et al. (2022)	Integrative review	Nurses and comprehensive geriatric assessment	PROSPERO CRD42021229953	Dual selection, single extraction	NIH, CASP and MMAT tools	U-PROFIT reports (Bleijenberg et al., 2016; 2017)
Manuel et al. (2025)	Systematic review	Hospital implementation of comprehensive geriatric assessment	PROSPERO CRD42022360172	Dual selection and dual extraction	NIH quality tools	None
Del Moral-Pairada et al. (2025)	Systematic review	Effectiveness of comprehensive geriatric assessment	PROSPERO CRD42023459782	Dual selection, extraction and quality assessment	QualSyst	U-PROFIT reports (Bleijenberg et al., 2016; 2017)
Fulceri et al. (2026)	Systematic review	Effectiveness of comprehensive geriatric assessment	PROSPERO CRD42024562519	Dual selection and extraction	Jadad scale	None
Azzellino et al. (2026b)	Scoping review	Nursing care models for older adults with frailty	Open Science Framework	JBI scoping approach	None reported	None
Choi et al. (2023)	Scoping review	Comprehensive geriatric assessment in older adults	Not reported	Not reported	Not reported	None
Guan et al. (2025)	Systematic review	Interventions to improve frailty among community-dwelling older adults	Not reported	Not reported	Not reported	None
Xu et al. (2024)	Systematic review with meta-analysis	Comprehensive geriatric assessment in hospital settings	Not reported	Not reported	Not reported	None
Chen et al. (2026)	Scoping review	Telemedicine-supported nursing care for older adults	Not reported	Not reported	Not reported	None
Antunes et al. (2023)	Qualitative synthesis	Rehabilitation nursing and frailty	Not reported	Not reported	Not reported	None

**Table 4:** Number of studies contributing to each theme.

**Theme**	Primary studies (n = 23)	**Evidence syntheses (n** **=** **12)**
1. Typology of multicomponent nursing interventions	23	12
2. Frailty outcomes and reported changes in frailty status	23	5
3. The nurse's role and competencies	23	9
4. Implementation barriers and facilitators	10	5
5. Geographic distribution of the evidence	23	4

## 4 Discussion

### 4.1 Principal findings

This integrative review re-adjudicated and re-characterised the evidence on nurse-led, nurse-delivered and nurse-coordinated multicomponent interventions for frailty in older adults using full texts, explicit operational criteria for frailty status and nursing involvement, and a separate analytic corpus of 23 primary studies. A consistent typology emerged: exercise-based multicomponent programmes combining structured physical activity with nutrition, education and psychosocial support, frequently embedded in comprehensive geriatric assessment pathways. The outcome evidence was more modest than the broader review literature suggests. One randomised trial reported a significant between-group categorical transition toward robust status (Yas and Secginli, 2025), three reported significant between-group improvements in validated continuous frailty scores (Luo et al., 2024; Huang et al., 2025; Ha and Park, 2020) and two quasi-experimental studies improved composite or non-validated frailty scores (Song and Boo, 2022; Lee et al., 2025), while one pilot trial found no significant between-group effect on frailty index change (Kim et al., 2025), one trial reported only within-group improvements (Faria et al., 2023) and one non-randomised study reported descriptive improvement without between-group testing (Zhou et al., 2025); four further studies reported within-group improvements without between-group testing, and one additional randomised trial found no between-group difference in daily functioning (van Lieshout et al., 2018). Ten of the 23 studies did not assess frailty status at all. The available evidence therefore shows promising associations between multicomponent nursing interventions and improvements in some frailty-related outcomes, most consistently for pre-frail community-dwelling older adults, but it does not establish that nursing interventions reverse frailty trajectories, and no pooled effect estimates are available.

### 4.2 Comparison with the wider literature

Our typology is concordant with international guidance favouring multicomponent management of physical frailty (Dent et al., 2019) and with the WHO Integrated Care for Older People framework's emphasis on integrated, person-centred care targeting intrinsic capacity (World Health Organization, 2019; Cesari et al., 2018). A recent systematic review of nurse-led interventions for pre-frail or frail older adults reported improvements in physical and mental health outcomes (Zheng et al., 2024), consistent with the pattern we observed while underscoring, as we do, the heterogeneity of instruments and outcomes. The prominence of implementation determinants in our corpus, including fidelity decay, integration difficulty and uneven component delivery (Bleijenberg et al., 2016; Muntinga et al., 2015; Ettema et al., 2015), echoes the implementation findings of the retained syntheses (Manuel et al., 2025; Azzellino et al., 2026b).

### 4.3 Strengths and limitations

Strengths of this revision include full-text-based re-adjudication of every previously included study against explicit operational criteria; a redesigned and re-executed search whose revised PubMed component retrieved all four corrected known-item validation items and whose 13,663 retrieved records were screened in full; a corrected and documented citation-level reconciliation of all counts across tables and figures; design-specific MMAT appraisal without composite scores; AMSTAR 2 appraisal of syntheses; a documented overlap matrix separating primary from review-level evidence; and prospective protocol registration with all deviations reported. Several limitations remain. First, 23 potentially eligible reports identified at abstract level could not be retrieved as full texts despite retrieval attempts via repositories, author manuscripts and DOI resolution, and six further evidence syntheses identified in the updated search await full-text assessment; both groups are documented in Supplementary Table S4. Second, the EBSCOhost and SpringerLink searches were used as verification sources and were not screened at record level, and Scopus, CINAHL and Embase were not re-searched with the revised strategy, although parallel strings are provided in Supplementary Table S5 for execution through institutional access. Third, two Chinese-language reports could not be obtained as full texts and are documented but not analysed. Fourth, four syntheses were appraised from abstracts and registration records. Fifth, data extraction and coding were performed by one reviewer with independent verification of a 20% sample, not full dual extraction, and per-record dual-screening decisions were not retained, so chance-corrected agreement statistics could not be reported. Sixth, the restriction to publications from 2015 onward excludes earlier eligible studies. Seventh, the corpus is dominated by high-income country settings, limiting generalisability. No meta-analysis was possible given the heterogeneity of designs, instruments and outcomes.

### 4.4 Implications for practice, policy and research

For practice, the most promising programmes for pre-frail community-dwelling older adults combine exercise with nutrition and education, are delivered or coordinated by nurses over at least 12 weeks, and include explicit follow-up; programme designers should attend to the fidelity problems documented here, particularly the under-delivery of nutrition components and the decay of assessment adherence over time. For policy, nursing roles in comprehensive geriatric assessment and case management are consistent with the WHO Integrated Care for Older People approach, although the economic evidence base remains sparse and predominantly European. For research, adequately powered randomised trials with categorical frailty status as a primary outcome, pre-registered protocols, validated instruments and transparent reporting of between-group estimates with confidence intervals are needed, particularly in low- and middle-income country settings, where the evidence gap is most pronounced.

## 5 Conclusion

This integrative review of 23 primary studies and 12 evidence syntheses found that nurse-led, nurse-delivered and nurse-coordinated multicomponent interventions show promising associations with improvements in some frailty-related outcomes in controlled studies, most consistently for pre-frail community-dwelling older adults, but that the available evidence does not establish that nursing interventions reverse frailty trajectories. Reports of improvement in frailty status rest on a small number of trials with heterogeneous instruments, and ten of the 23 primary studies did not assess frailty status at all. Effect estimates have not been pooled. Evidence from low- and middle-income countries remains very limited. Adequately powered randomised trials with categorical frailty status as a primary outcome, conducted across diverse settings including low- and middle-income countries, are required to determine whether multicomponent nursing interventions can change frailty trajectories.

## Ethics approval and consent to participate

Not applicable. This review analysed published literature only.

## Consent for publication

Not applicable.

## Availability of data and materials

All data generated or analysed during this review are included in this article and its supplementary files, including the search strings, screening records, extraction tables, appraisal ratings and citation matrix.

## Use of artificial intelligence tools

The authors used an AI-assisted research agent to support database searching, record-level screening of the updated search, full-text retrieval, bibliographic validation of citations against CrossRef and PubMed, and drafting of the revised manuscript. All study selection decisions, re-adjudication judgments, data extraction, methodological appraisals, interpretations and final wording were reviewed, verified and approved by the authors, who take full responsibility for the content. Every citation was validated against CrossRef and PubMed records; no AI tool was used to generate data, study results or references. The revised search strings, record counts and validation results reported in Section 2.2 are documented in the supplementary files.

## Declaration of generative AI and AI-assisted technologies

During the preparation of this work the author(s) used an AI-assisted research and document-preparation tool to support reference parsing, de-duplication, screening-log organisation and drafting/formatting of the manuscript and supplementary tables. After using this tool, the author(s) reviewed and edited the content as needed and take full responsibility for the content of the publication. No generative AI was used to fabricate, alter or invent data, results or references.

## Funding

This publication charge is funded by Unpad through the Indonesian Endowment Fund for Education (LPDP) on behalf of the Indonesian Ministry of Higher Education, Science and Technology and managed under the EQUITY Program (Contract No 4303/ B3/DT.03.08/2025 and 3927/UN6. RKT/HK.07.00/2025). The funder had no role in study design, data collection and analysis, decision to publish, or manuscript preparation.

Registration: Open Science Framework, DOI 10.17605/OSF.IO/B4RCJ, registered 10 December 2025.

## CRediT authorship contribution statement

**Dhika Dharmansyah:** Writing – review & editing, Writing – original draft, Methodology, Investigation, Formal analysis, Data curation, Conceptualization. **Laili Rahayuwati:** Writing – review & editing, Validation, Supervision, Methodology, Conceptualization. **Iqbal Pramukti:** Writing – review & editing, Validation, Methodology, Formal analysis. **Kuswandewi Mutyara:** Writing – review & editing, Validation, Methodology. **Sehabudin Salasa:** Writing – review & editing, Investigation, Data curation.

## Declaration of competing interest

The authors declare the following financial interests/personal relationships which may be considered as potential competing interests: Dhika Dharmansyah reports financial support was provided by Padjadjaran University. Reports a relationship with that includes:. Has patent pending to. If there are other authors, they declare that they have no known competing financial interests or personal relationships that could have appeared to influence the work reported in this paper.

## Data Availability

The review protocol, full search strategy, screening log and supporting materials are openly available on the Open Science Framework (https://doi.org/10.17605/OSF.IO/B4RCJ). Supplementary tables (S1 characteristics; S2 MMAT appraisal template; S3 synthesis matrix; S4 screening log) accompany this manuscript.
