# Supplementary material for: Multicomponent nursing interventions and frailty-related outcomes in older adults: An integrative review of primary studies and evidence syntheses
Source: Int J Nurs Stud Adv. 2026 Oct 1;11:100687. doi: 10.1016/j.ijnsa.2026.100687 (PMC13675801; doi:10.1016/j.ijnsa.2026.100687)
Supplement: Supplementary file 1 [file mmc1.docx]

**Supplementary Table S7**

*Citation matrix: overlap between the 12 retained evidence syntheses and the 14 primary studies retained from the original search*

Manuscript IJNSA-D-26-00510R1. Multicomponent Nursing Interventions and Frailty-Related Outcomes in Older Adults: An Integrative Review of Primary Studies and Evidence Syntheses.

X indicates that the primary study was included in the corresponding evidence synthesis, as reported in the synthesis reference lists. Overlap was limited: Del Moral-Pairada et al. (2025) and Sum et al. (2022) included reports from the U-PROFIT programme (Bleijenberg et al. 2016; 2017), and Kasa et al. (2023) included Song and Boo (2022). No other study retained from the original search was included in any retained synthesis.

| **Synthesis \ Primary study** | **Faria 2023** | **Yas 2025** | **Kim 2025** | **Luo 2024** | **Huang 2025** | **Zhou 2025** | **Song & Boo 2022** | **Bleijenberg 2017** | **Bleijenberg 2016** | **Muntinga 2015** | **Ettema 2015** | **Azzellino 2026a** | **Ron 2025** | **Cypel 2026** |
| --- | --- | --- | --- | --- | --- | --- | --- | --- | --- | --- | --- | --- | --- | --- |
| Kasa 2023 |  |  |  |  |  |  | X |  |  |  |  |  |  |  |
| Morkisch 2020 |  |  |  |  |  |  |  |  |  |  |  |  |  |  |
| Sum 2022 |  |  |  |  |  |  |  | X | X |  |  |  |  |  |
| Manuel 2025 |  |  |  |  |  |  |  |  |  |  |  |  |  |  |
| Del Moral 2025 |  |  |  |  |  |  |  | X | X |  |  |  |  |  |
| Fulceri 2026 |  |  |  |  |  |  |  |  |  |  |  |  |  |  |
| Azzellino 2026b |  |  |  |  |  |  |  |  |  |  |  |  |  |  |
| Choi 2023 |  |  |  |  |  |  |  |  |  |  |  |  |  |  |
| Guan 2025 |  |  |  |  |  |  |  |  |  |  |  |  |  |  |
| Xu 2024 |  |  |  |  |  |  |  |  |  |  |  |  |  |  |
| Chen 2026 |  |  |  |  |  |  |  |  |  |  |  |  |  |  |
| Antunes 2023 |  |  |  |  |  |  |  |  |  |  |  |  |  |  |

*Backward citation searching of all retained syntheses' reference lists and forward citation searching of the retained primary studies identified no additional primary study meeting all revised eligibility criteria.*

*The nine studies added through the updated PubMed search (Supplementary Table S1, Section B) were identified by database searching rather than citation searching and were integrated into the analytic corpus, which now comprises 23 primary studies. Their containment within the retained syntheses was not re-assessed in this revision because most syntheses were available only as abstracts and registration records (Supplementary Table S8); the matrix above therefore documents the verified overlap for the 14 studies retained from the original search.*
