# Supplementary material for: Multicomponent nursing interventions and frailty-related outcomes in older adults: An integrative review of primary studies and evidence syntheses
Source: Int J Nurs Stud Adv. 2026 Oct 1;11:100687. doi: 10.1016/j.ijnsa.2026.100687 (PMC13675801; doi:10.1016/j.ijnsa.2026.100687)
Supplement: Supplementary file 2 [file mmc2.docx]

**Supplementary Table S3**

*Quality assurance and agreement documentation for study screening and selection*

Manuscript IJNSA-D-26-00510R1. Multicomponent Nursing Interventions and Frailty-Related Outcomes in Older Adults: An Integrative Review of Primary Studies and Evidence Syntheses.

This supplement documents the dual-review quality-assurance procedures used at each screening and selection stage and explains transparently why inter-rater agreement statistics cannot be reported for this revision.

## **Part 1. Dual-review quality-assurance procedures**

| **Stage** | **Reviewers** | **Scope of dual review** | **Discrepancy resolution** |
| --- | --- | --- | --- |
| Title/abstract screening, original search (15 January - 12 February 2026) | R1 (all records) + R2 (20% random sample independently double-screened) | All 273 records screened by R1; a 20% random sample independently re-screened by R2 | Discussion, with arbitration by a second reviewer |
| Full-text re-adjudication of the 32 previously eligible reports (1 - 10 September 2026) | R1 + R2 (all reports) | Independent adjudication of every report against the revised operational criteria | Discussion and arbitration; final decisions recorded in Supplementary Table S6 |
| Title/abstract screening, updated PubMed search (September 2026) | R1 (all 13,411 records) | Automated removal of 252 ineligible publication types followed by title/abstract screening; all candidate reports retrieved and assessed at full text | Ambiguous records retained for full-text assessment rather than excluded |

## **Part 2. Why agreement statistics are not reported**

Percentage agreement and Cohen's kappa require paired per-record decisions from both reviewers at the same screening stage. The per-record dual-screening decision log from the original title/abstract screening (February 2026) was not retained in a form that allows paired decisions to be reconstructed: the screening export preserved only the reconciled final decision per record, together with the sampling frame and resolution notes. Agreement statistics therefore cannot be computed without reconstructing reviewer decisions that were not documented at the time, which would not constitute a genuine record of the screening process and is not reported as such.

In place of statistics that cannot be computed honestly, this revision reports: (a) the dual-review procedures documented in Part 1, including 20% independent double-screening of the original search and full dual re-adjudication of all 32 previously eligible reports; (b) the complete re-adjudication record for every previously included study (Supplementary Table S6); (c) the screening log of the updated search with per-record decisions and exclusion codes (available on request; summary counts in Supplementary Table S4); and (d) a manuscript text (Section 2.3) that describes the quality-assurance procedures actually used rather than promising a kappa statistic. For completeness, the formulas that would apply to paired decisions, had they been retained, are recorded below.

Cohen's kappa is computed as kappa = (Po - Pe) / (1 - Pe), where Po is the observed proportion of agreement and Pe the proportion expected by chance; interpretations follow Landis and Koch (0.00-0.20 slight; 0.21-0.40 fair; 0.41-0.60 moderate; 0.61-0.80 substantial; 0.81-1.00 almost perfect). Percentage agreement = n agreements / n double-screened x 100.
