# Supplementary material for: Multicomponent nursing interventions and frailty-related outcomes in older adults: An integrative review of primary studies and evidence syntheses
Source: Int J Nurs Stud Adv. 2026 Oct 1;11:100687. doi: 10.1016/j.ijnsa.2026.100687 (PMC13675801; doi:10.1016/j.ijnsa.2026.100687)
Supplement: Supplementary file 3 [file mmc3.docx]

**Supplementary Table S5**

*Complete search strings and per-database record counts*

Manuscript IJNSA-D-26-00510R1. Multicomponent Nursing Interventions and Frailty-Related Outcomes in Older Adults: An Integrative Review of Primary Studies and Evidence Syntheses.

## **Part 1. Per-database summary**

| **Database / interface** | **Strategy** | **Date executed** | **Records retrieved** |
| --- | --- | --- | --- |
| PubMed (NLM) | Original two-block strategy ( frailty terms; multicomponent intervention terms ), 2015 onward | 15 January 2026 | 46 |
| Scopus (Elsevier) | Original strategy, translated | 15 January 2026 | 270 |
| PubMed (NLM) | Revised strategy (final string, Part 2) | 20 September 2026 | 13,663 |
| EBSCOhost (EBSCO academic research interface) | Revised strategy, CINAHL translation (Part 4) with date limiter 20150101-20260920; verification search | 20 September 2026 | 8,398 |
| SpringerLink (Springer Nature) | Revised strategy, translated, date facets 2015-2026; verification search | 20 September 2026 | 40,614 |
| Scopus (Elsevier) | Revised strategy (Part 3) | Not executed in this revision | - |
| CINAHL (EBSCO) | Revised strategy (Part 4) | Not executed in this revision | - |
| Embase (Elsevier) | Revised strategy (Part 5) | Not executed in this revision | - |

Validation: the revised PubMed strategy retrieved all four items of the corrected known-item validation set (Zheng et al. 2024, Ageing Research Reviews; Kasa et al. 2023; Song and Boo 2022; Yas and Secginli 2025). The EBSCOhost and SpringerLink searches (20 September 2026) were executed as verification searches for records not indexed in PubMed: the top-ranked results of both searches were reviewed and no additional eligible primary study was apparent; neither search was screened at record level, and both are reported as verification rather than exhaustive screening sources (manuscript Sections 2.2 and 4.3). The Scopus, CINAHL and Embase strings below are final and ready to run without modification through Universitas Padjadjaran institutional access.

## **Part 2. Revised PubMed strategy (final; executed 20 September 2026; 13,663 records)**

("Frailty"[Mesh] OR frailty[tiab] OR frail[tiab] OR prefrailty[tiab] OR prefrail[tiab] OR "pre-frailty"[tiab] OR "pre-frail"[tiab] OR "pre frailty"[tiab] OR "pre frail"[tiab] OR "frailty syndrome"[tiab] OR "at risk of frailty"[tiab])

**AND**

(multicomponent[tiab] OR "multi-component"[tiab] OR "comprehensive geriatric assessment"[tiab] OR "geriatric assessment"[tiab] OR multidimensional[tiab] OR multidisciplinary[tiab] OR "transitional care"[tiab] OR "case management"[tiab] OR "integrated care"[tiab] OR prehabilitation[tiab] OR "nurse-led"[tiab] OR "nursing intervention"[tiab] OR "educational intervention"[tiab] OR "care coordination"[tiab] OR exercise[tiab] OR exercises[tiab] OR "physical activity"[tiab] OR "resistance training"[tiab] OR "strength training"[tiab] OR "balance training"[tiab] OR "physical training"[tiab] OR nutrition[tiab] OR nutritional[tiab] OR "dietary intervention"[tiab] OR "nutritional intervention"[tiab] OR "oral nutritional supplement"[tiab] OR "protein supplementation"[tiab])

AND 2015/01/01:2026/09/20[dp]

## **Part 3. Revised Scopus strategy**

TITLE-ABS-KEY(( frailty OR frail OR prefrailty OR prefrail OR "pre-frailty" OR "pre-frail" OR "pre frailty" OR "pre frail" OR "frailty syndrome" OR "at risk of frailty" )) AND TITLE-ABS-KEY(( multicomponent OR "multi-component" OR "comprehensive geriatric assessment" OR "geriatric assessment" OR multidimensional OR multidisciplinary OR "transitional care" OR "case management" OR "integrated care" OR prehabilitation OR "nurse-led" OR "nursing intervention" OR "educational intervention" OR "care coordination" OR exercise OR exercises OR "physical activity" OR "resistance training" OR "strength training" OR "balance training" OR "physical training" OR nutrition OR nutritional OR "dietary intervention" OR "nutritional intervention" OR "oral nutritional supplement" OR "protein supplementation" )) AND PUBYEAR > 2014

## **Part 4. Revised CINAHL (EBSCO) strategy**

((MH "Frailty" OR MH "Frail Elderly") OR TI ( frailty OR frail OR prefrailty OR prefrail OR "pre-frailty" OR "pre-frail" OR "frailty syndrome" OR "at risk of frailty" ) OR AB ( frailty OR frail OR prefrailty OR prefrail OR "pre-frailty" OR "pre-frail" OR "frailty syndrome" OR "at risk of frailty" )) AND ((MH "Geriatric Assessment") OR TI ( multicomponent OR "multi-component" OR "comprehensive geriatric assessment" OR "geriatric assessment" OR multidimensional OR multidisciplinary OR "transitional care" OR "case management" OR "integrated care" OR prehabilitation OR "nurse-led" OR "nursing intervention" OR "educational intervention" OR "care coordination" OR exercise OR exercises OR "physical activity" OR "resistance training" OR "strength training" OR "balance training" OR "physical training" OR nutrition OR nutritional OR "dietary intervention" OR "nutritional intervention" OR "oral nutritional supplement" OR "protein supplementation" ) OR AB ( multicomponent OR "multi-component" OR "comprehensive geriatric assessment" OR "geriatric assessment" OR multidimensional OR multidisciplinary OR "transitional care" OR "case management" OR "integrated care" OR prehabilitation OR "nurse-led" OR "nursing intervention" OR "educational intervention" OR "care coordination" OR exercise OR exercises OR "physical activity" OR "resistance training" OR "strength training" OR "balance training" OR "physical training" OR nutrition OR nutritional OR "dietary intervention" OR "nutritional intervention" OR "oral nutritional supplement" OR "protein supplementation" ))

Limiters: Published Date: 20150101-20260920

## **Part 5. Revised Embase strategy**

('frailty'/exp OR 'frail elderly'/exp OR frailty:ti,ab OR frail:ti,ab OR prefrailty:ti,ab OR prefrail:ti,ab OR 'pre-frailty':ti,ab OR 'pre-frail':ti,ab OR 'frailty syndrome':ti,ab OR 'at risk of frailty':ti,ab) AND ('comprehensive geriatric assessment'/exp OR 'geriatric assessment':ti,ab OR multicomponent:ti,ab OR 'multi-component':ti,ab OR multidimensional:ti,ab OR multidisciplinary:ti,ab OR 'transitional care':ti,ab OR 'case management':ti,ab OR 'integrated care':ti,ab OR prehabilitation:ti,ab OR 'nurse-led':ti,ab OR 'nursing intervention':ti,ab OR 'educational intervention':ti,ab OR 'care coordination':ti,ab OR exercise:ti,ab OR exercises:ti,ab OR 'physical activity':ti,ab OR 'resistance training':ti,ab OR 'strength training':ti,ab OR 'balance training':ti,ab OR 'physical training':ti,ab OR nutrition:ti,ab OR nutritional:ti,ab OR 'dietary intervention':ti,ab OR 'nutritional intervention':ti,ab OR 'oral nutritional supplement':ti,ab OR 'protein supplementation':ti,ab) AND [2015-2026]/py

*Design note: the revised strategy removes the requirement for nursing-role terminology (assessed instead at full-text appraisal), separates the exercise and nutrition concepts with Boolean OR, and broadens the frailty free-text set, as described in manuscript Section 2.2. Verification searches were additionally executed in EBSCOhost (8,398 records) and SpringerLink (40,614 records) on 20 September 2026.*
