# Supplementary material for: Multicomponent nursing interventions and frailty-related outcomes in older adults: An integrative review of primary studies and evidence syntheses
Source: Int J Nurs Stud Adv. 2026 Oct 1;11:100687. doi: 10.1016/j.ijnsa.2026.100687 (PMC13675801; doi:10.1016/j.ijnsa.2026.100687)
Supplement: Supplementary file 4 [file mmc4.docx]

**Supplementary Table S4**

*Exclusion codes, decision rules and record counts for the updated PubMed search (20 September 2026)*

Manuscript IJNSA-D-26-00510R1. Multicomponent Nursing Interventions and Frailty-Related Outcomes in Older Adults: An Integrative Review of Primary Studies and Evidence Syntheses.

## **Part 1. Flow counts**

| **Stage** | **n** |
| --- | --- |
| Records identified (PubMed, revised strategy, 2015/01/01 to 2026/09/20) | 13,663 |
| Records removed before screening: marked as ineligible by automation tools (publication type filters: letters, editorials, comments, news, case reports, practice guidelines) | 252 |
| Records screened at title/abstract level | 13,411 |
| Records excluded at title/abstract level (code E2, E3, E5-E9) | 13,342 |
| Records that were reports of studies already included in the review | 25 |
| Reports sought for retrieval | 44 |
| Reports not retrieved (paywalled; code E10) | 23 |
| Reports assessed for eligibility | 21 |
| New studies included and integrated into the analytic corpus | 9 |
| Reports excluded at full-text assessment (codes E11-E14) | 12 |

## **Part 2. Exclusion codes**

| **Code** | **Definition** |
| --- | --- |
| E1 | Publication type ineligible (letter, editorial, comment, news, case report, practice guideline) - removed before screening by automated filters |
| E2 | No frailty, pre-frailty or validated frailty-risk criterion documented (title/abstract level) |
| E3 | No nursing involvement documented (title/abstract level) |
| E5 | Single-component intervention (exercise, nutrition or education alone) |
| E6 | Population outside scope (not older adults, or age range including < 65 without frailty focus) |
| E7 | Outcome outside scope (no frailty-related or functional outcome) |
| E8 | Review or synthesis with no primary data and outside synthesis eligibility |
| E9 | Other: protocol, design/development or feasibility-protocol paper; qualitative-only design; population not frailty-defined |
| E10 | Full text not retrieved (paywalled despite retrieval attempts via open-access repositories, author manuscripts, thesis repositories and DOI resolution) |
| E11 | No nursing involvement documented at full-text assessment |
| E12 | No frailty or pre-frailty eligibility criterion at full-text assessment |
| E13 | Companion report of an already-represented trial (duplicate publication) |
| E14 | Provisional exclusion pending final author adjudication (documented reason in Part 4) |
| ALREADY | Report of a study already included in the review corpus |

## **Part 3. Reports excluded at full-text assessment (n = 12)**

| **Report (PMID)** | **Code** | **Reason** |
| --- | --- | --- |
| Naughton et al. 2024, Frailty Care Bundle, J Frailty Sarcopenia Musc (38444547) | E12 | No frailty/prefrailty eligibility criterion at baseline (inclusion: age 60+, medically stable, mobile pre-admission); cognitive impairment excluded |
| SAFE transitional care unit, Canada (39619381) | E11 | Nursing involvement not documented; interdisciplinary geriatric restorative unit; no explicit frailty instrument at admission |
| SPEC stepped-wedge technology-enhanced care model (32668005) | E11 | Nursing role not documented (facility-level care model); no individual-level frailty criterion |
| Intelligent exercise system, China (42661865) | E11 | Nursing involvement not documented; implementation-focused evaluation |
| Multicomponent Otago exercise with resistance training, China (41246478) | E11 | No nursing involvement (rehabilitation therapists); primary outcome sarcopenia; exercise-only programme |
| CCB process evaluation (33594695) | E13 | Companion process evaluation of the CCB trial; main trial report included (Jepma et al. 2021) |
| CCB qualitative study (34372851) | E13 | Companion qualitative study of the CCB trial |
| CCB economic evaluation (35085361) | E13 | Companion economic evaluation of the CCB trial |
| U-PROFIT effectiveness report, JAGS (27459236) | E13 | Companion effectiveness report of the U-PROFIT trial already represented in the corpus (Bleijenberg et al. 2016, 2017) |
| Di Pollina et al. 2017, Geneva integrated care (28196486) | E14 | Provisional exclusion pending author adjudication: nursing role partial (community geriatrics unit with nursing teams) |
| Tam et al. 2025, ICAH Hong Kong (40485272) | E14 | Provisional exclusion pending author adjudication: very advanced frailty (Clinical Frailty Scale >= 7, median 8) - scope decision |
| Exercise with nursing interventions, China (40485761) | E14 | Provisional exclusion pending author adjudication: serious methodological concerns (retrospective design with claimed randomisation) |

## **Part 4. Reports not retrieved (n = 23; potentially eligible at abstract level)**

| **Report (PMID)** | **Provisional assessment** |
| --- | --- |
| Kim et al. 2025, N-POWER, Geriatr Nurs (40616927) | RCT n = 41, pre-frail older women; nurse-led exercise + nutrition + cognitive coaching; frailty improved |
| Taiwan FPCMP, Geriatr Nurs (42241906) | Quasi-experimental; nurse-led TRX exercise + nutrition + self-management; frailty outcome |
| Wang et al. 2025, perioperative nursing RCT, Clin Rehabil (40255050) | RCT n = 300; frail cardiac surgery patients; comprehensive perioperative nursing |
| Spain outpatient CGA + exercise (39947016) | Observational; frail/pre-frail (SPPB); multicomponent with specialised nursing team |
| Nurse-led CGA feasibility cRCT, JAN (37002595) | Feasibility cluster RCT; moderately/severely frail; nurse-led CGA |
| New Zealand RN-led proactive care, JAGS (33629356) | Quasi-experiment n = 1,085 vs 3,750; RN-led CGA + care planning; frailty criterion to verify |
| Blom et al. 2016, CareWell, J Am Board Fam Med (26957377) | Cluster controlled trial n = 536; frail >= 70 (EASY-Care); GP-led with practice/community nurse case management - nursing role partial |
| Denmark transitional care RCT, Age Ageing (34479071) | RCT n = 3,103; frail >= 75 acutely admitted; municipality nurse-led transitional care |
| AGe-FIT long-term evaluation, JAMDA (26805750) | RCT; outpatient CGA as complement to usual care; nursing role to verify |
| Spanish primary care nursing pilot, Enferm Clin (42476315) | Pilot RCT n = 24; primary care nursing intervention for frailty; frailty improved |
| GEDI model, Emerg Med Australas (31773885) | Non-randomised; nurse-led emergency department model; frailty criterion to verify |
| Japanese preventive home visits, J Gerontol A (27075895) | RCT n = 360; home visits by nurses or care managers - nursing role mixed |
| Swedish case management RCT, Health Soc Care Community (28295847) | RCT n = 153; case management by nurses and physiotherapists; social participation outcomes |
| Brisbane ED nurse-led model, Aging Clin Exp Res (30617858) | Pre-post n = 1,130; all frail (Clinical Frailty Scale) |
| MILES nurse-led perioperative service, Singapore (40669134) | Retrospective n = 949; design and frailty criterion to verify |
| ITHBC nursing intervention T2DM, Res Gerontol Nurs (39589097) | Cluster RCT n = 71; 12-week theory-based nursing intervention; components to verify |
| Cognitive-motor dual-task training, Geriatr Nurs (39742543) | Quasi-experimental n = 65; cognitive frailty population - construct scope decision |
| Six evidence syntheses (38951122; 42675567; 32200100; 38920437; 40308686; 42547949) | Systematic reviews and meta-analyses of nurse-coordinated/nurse-led interventions; full-text assessment pending |

*Note. The full screening log (per-record decisions with PMIDs) is held by the authors and is available on request. Reports with code E10 remain formally unresolved for this revision; the updated PRISMA flow (Figure 1) reports them under 'reports not retrieved'.*
