# Supplementary material for: Multicomponent nursing interventions and frailty-related outcomes in older adults: An integrative review of primary studies and evidence syntheses
Source: Int J Nurs Stud Adv. 2026 Oct 1;11:100687. doi: 10.1016/j.ijnsa.2026.100687 (PMC13675801; doi:10.1016/j.ijnsa.2026.100687)
Supplement: Supplementary file 5 [file mmc5.docx]

**Supplementary Table S8**

*Appraisal of the 12 retained evidence syntheses (AMSTAR 2 domains)*

Manuscript IJNSA-D-26-00510R1. Multicomponent Nursing Interventions and Frailty-Related Outcomes in Older Adults: An Integrative Review of Primary Studies and Evidence Syntheses.

Ratings are reported for the AMSTAR 2 items that are verifiable from the syntheses' published reports and registration records: prospective protocol registration (item 2), independent dual study selection (item 4), duplicate data extraction (item 5), and use of an appropriate risk-of-bias / critical-appraisal technique (item 9). Seven syntheses reported prospective registration (six PROSPERO, one Open Science Framework); five did not. For four syntheses available only as paywalled full texts, appraisal relied on abstracts and registration records, and the corresponding ratings should be interpreted with caution; per-item responses for the remaining AMSTAR 2 items are held in the authors' appraisal records and are available on request.

| **Synthesis** | **Design** | **Protocol registration (item 2)** | **Dual selection (item 4)** | **Dual extraction (item 5)** | **Risk-of-bias / appraisal technique (item 9)** | **Overlap with primary corpus** | **Appraisal basis** |
| --- | --- | --- | --- | --- | --- | --- | --- |
| Kasa et al. (2023) | Systematic review | Yes (PROSPERO CRD42022348064) | Yes | Yes | Reported (tool not specified in available records) | None | Full text |
| Morkisch et al. (2020) | Systematic review | Yes (PROSPERO CRD42018084604) | Yes | Yes | Cochrane risk-of-bias tool | None | Full text |
| Sum et al. (2022) | Integrative review | Yes (PROSPERO CRD42021229953) | Yes | No (single extraction) | NIH, CASP and MMAT tools | U-PROFIT reports (Bleijenberg 2016; 2017) | Full text |
| Manuel et al. (2025) | Systematic review | Yes (PROSPERO CRD42022360172) | Yes | Yes | NIH quality tools | None | Full text |
| Del Moral-Pairada et al. (2025) | Systematic review | Yes (PROSPERO CRD42023459782) | Yes | Yes | QualSyst | U-PROFIT reports (Bleijenberg 2016; 2017) | Full text |
| Fulceri et al. (2026) | Systematic review | Yes (PROSPERO CRD42024562519) | Yes | Yes | Jadad scale | None | Full text |
| Azzellino et al. (2026b) | Scoping review | Yes (Open Science Framework) | Yes | Not reported | None reported | None | Paywalled - abstract and registration record |
| Choi et al. (2023) | Scoping review | No | Not reported | Not reported | Not reported | None | Paywalled - abstract |
| Guan et al. (2025) | Systematic review | No | Not reported | Not reported | Not reported | None | Paywalled - abstract |
| Xu et al. (2024) | Systematic review with meta-analysis | No | Not reported | Not reported | Not reported | None | Paywalled - abstract |
| Chen et al. (2026) | Scoping review | No | Not reported | Not reported | Not reported | None | Paywalled - abstract |
| Antunes et al. (2023) | Qualitative synthesis | No | Not reported | Not reported | Not reported | None | Paywalled - abstract |

*Note. AMSTAR 2 = A Measurement Tool to Assess Systematic Reviews. No overall confidence rating is assigned here because per-item responses across all 16 AMSTAR 2 items are required for a defensible overall judgement; the completed item-level checklists are held in the authors' appraisal records.*
