# Supplementary material for: Multicomponent nursing interventions and frailty-related outcomes in older adults: An integrative review of primary studies and evidence syntheses
Source: Int J Nurs Stud Adv. 2026 Oct 1;11:100687. doi: 10.1016/j.ijnsa.2026.100687 (PMC13675801; doi:10.1016/j.ijnsa.2026.100687)
Supplement: Supplementary file 6 [file mmc6.docx]

**Supplementary Table S6**

*Full-text re-adjudication of the 32 previously eligible reports against the revised operational criteria*

Manuscript IJNSA-D-26-00510R1. Multicomponent Nursing Interventions and Frailty-Related Outcomes in Older Adults: An Integrative Review of Primary Studies and Evidence Syntheses.

Re-adjudication was performed between 1 and 10 September 2026 by two reviewers working independently, with disagreements resolved through discussion and arbitration, against the revised operational criteria (documented frailty, pre-frailty or validated frailty-risk criterion; defined minimum nursing involvement). Twenty-six reports were retained (14 primary studies; 12 evidence syntheses) and six were excluded. The nine studies subsequently identified through the updated PubMed search (20 September 2026) and integrated into the analytic corpus are documented separately in Supplementary Table S1, Section B; they were not part of this re-adjudication, which applied to the 32 previously eligible reports only.

| **Report** | **Category** | **Disposition** | **Reason** |
| --- | --- | --- | --- |
| Faria et al. (2023) | Primary | Retained | Meets all revised criteria |
| Yas and Secginli (2025) | Primary | Retained | Meets all revised criteria |
| Kim et al. (2025) | Primary | Retained | Meets all revised criteria |
| Luo et al. (2024) | Primary | Retained | Meets all revised criteria |
| Huang et al. (2025) | Primary | Retained | Meets all revised criteria; full text obtained and verified |
| Zhou et al. (2025) | Primary | Retained | Meets all revised criteria |
| Song and Boo (2022) | Primary | Retained | Meets all revised criteria |
| Bleijenberg et al. (2017) | Primary | Retained | Meets all revised criteria; full text obtained and verified |
| Bleijenberg et al. (2016) | Primary | Retained | Meets all revised criteria; full text obtained and verified |
| Muntinga et al. (2015) | Primary | Retained | Meets all revised criteria |
| Ettema et al. (2015) | Primary | Retained | Meets all revised criteria; full text obtained and verified |
| Azzellino et al. (2026a) | Primary | Retained | Meets all revised criteria |
| Ron et al. (2025) | Primary | Retained | Meets all revised criteria |
| Cypel et al. (2026) | Primary | Retained | Meets all revised criteria |
| Kasa et al. (2023) | Synthesis | Retained | Meets revised synthesis criteria |
| Morkisch et al. (2020) | Synthesis | Retained | Meets revised synthesis criteria |
| Sum et al. (2022) | Synthesis | Retained | Meets revised synthesis criteria |
| Manuel et al. (2025) | Synthesis | Retained | Meets revised synthesis criteria |
| Del Moral-Pairada et al. (2025) | Synthesis | Retained | Meets revised synthesis criteria |
| Fulceri et al. (2026) | Synthesis | Retained | Meets revised synthesis criteria |
| Azzellino et al. (2026b) | Synthesis | Retained | Meets revised synthesis criteria |
| Choi et al. (2023) | Synthesis | Retained | Meets revised synthesis criteria |
| Guan et al. (2025) | Synthesis | Retained | Meets revised synthesis criteria |
| Xu et al. (2024) | Synthesis | Retained | Meets revised synthesis criteria |
| Chen et al. (2026) | Synthesis | Retained | Meets revised synthesis criteria |
| Antunes et al. (2023) | Synthesis | Retained | Meets revised synthesis criteria |
| Alcolea-Ruiz et al. (2026) | Primary | Excluded | No frailty, pre-frailty or validated frailty-risk criterion documented: recruited by fear-of-falling status |
| Orcel et al. (2025) | Primary | Excluded | No frailty, pre-frailty or validated frailty-risk criterion documented: enrolled adults aged 70 years or older with chronic conditions |
| Irimia et al. (2022) | Primary | Excluded | No frailty, pre-frailty or validated frailty-risk criterion documented: service audit in which frailty was assessed only post hoc |
| Esser et al. (2026) | Primary | Excluded | No frailty, pre-frailty or validated frailty-risk criterion documented: heart failure population |
| Hongyan et al. (2022) | Primary | Excluded | Full text could not be obtained despite contact with authors and retrieval attempts; DOI does not resolve through CrossRef |
| Liang et al. (2023) | Primary | Excluded | Full text could not be obtained despite contact with authors and retrieval attempts; DOI does not resolve through CrossRef |
