# Supplementary material for: Multicomponent nursing interventions and frailty-related outcomes in older adults: An integrative review of primary studies and evidence syntheses
Source: Int J Nurs Stud Adv. 2026 Oct 1;11:100687. doi: 10.1016/j.ijnsa.2026.100687 (PMC13675801; doi:10.1016/j.ijnsa.2026.100687)
Supplement: Supplementary file 7 [file mmc7.docx]

**Supplementary Table S1**

*Data extraction table for primary studies included in the review and studies newly identified as eligible in the updated search*

Manuscript IJNSA-D-26-00510R1. Multicomponent Nursing Interventions and Frailty-Related Outcomes in Older Adults: An Integrative Review of Primary Studies and Evidence Syntheses.

## **Section A. Primary studies retained from the original search after re-adjudication (n = 14)**

Full texts of all retained primary studies were obtained during the revision. Extractions for the four studies available only as paywalled full texts at the time of first submission (Bleijenberg 2016, 2017; Ettema 2015; Huang 2025) were verified against full texts obtained from institutional access, publisher open-access records and the authors' doctoral thesis repositories; the verification note in the final column documents the source used for each verification.

| **Study (country)** | **Design** | **Population and frailty criterion** | **Nursing role** | **Intervention components** | **Frailty-related outcome** | **Reported result** | **Full-text verification** |
| --- | --- | --- | --- | --- | --- | --- | --- |
| Faria et al. (2023), Portugal | Randomised trial | Community-dwelling older adults with frailty (Tilburg Frailty Indicator) | Nurse-led | Home-based advanced practice rehabilitation nursing programme: exercise, education, psychosocial support (12 weeks) | Tilburg Frailty Indicator total and domain scores | Within-group improvements; no between-group test reported | Open access; extraction verified |
| Yas and Secginli (2025), Turkiye | Randomised trial | Pre-frail women aged 55 years or older (FRAIL scale), n = 84 | Nurse-led | Salutogenic programme: exercise, nutrition, education, psychosocial coaching (6 weeks) | FRAIL scale; frailty transitions | Significant between-group FRAIL improvement at 3, 6 and 9 months (p < 0.001); about half of the intervention group transitioned toward robust status | Open access; extraction verified |
| Kim et al. (2025), Republic of Korea | Pilot randomised trial | Adults aged 70 years or older with frailty (Korean Frailty Index for Primary Care >= 0.25), n = 84 | Nurse-delivered | Integrated care programme: exercise, nutrition, social support, care coordination | Korean Frailty Index for Primary Care | Group-by-time interaction significant (p = 0.009); between-group frailty index change non-significant at 3 and 6 months | Open access; extraction verified |
| Luo et al. (2024), China | Randomised trial | Older stroke patients with frailty (FRAIL scale score of 3 or more), n = 125 | Nurse-led | Multicomponent exercise nursing programme: progressive exercise, nutrition, education (12 weeks) | FRAIL scale | Significant between-group FRAIL differences at 4 and 12 weeks (both p < 0.001) | Open access; extraction verified |
| Huang et al. (2025), Taiwan | Randomised trial | Older adults after cardiac surgery, pre-frail or frail (Fried phenotype), n = 92 (46/46) | Nurse-delivered | Home-based multicomponent exercise with nutrition and nursing consultation (12 weeks) | Fried phenotype score | Significantly greater between-group frailty score improvement at 6 and 12 weeks | Full text verified via publisher (OUP) and published record: n = 92, 46/46, pre-frailty 67% / frailty 33% at baseline; NCT04332887 |
| Zhou et al. (2025), China | Non-randomised emulated target trial with propensity-score matching | Older adults after percutaneous coronary intervention (Fried-based frailty phenotype), n = 136 matched | Nurse-led | Seven-component digital cardiac rehabilitation: exercise, education, nutrition, remote follow-up | Frailty phenotype distribution; six-minute walk, gait speed, grip strength | Descriptive phenotype improvement (pre-frail 100% to 75%); baseline frailty imbalance between groups; no inferential between-group frailty test | Open access; extraction verified |
| Song and Boo (2022), Republic of Korea | Quasi-experimental (non-equivalent control group) | Community-dwelling older adults living alone with frailty (frailty score threshold) | Nurse-led | Multicomponent programme: exercise, nutrition, education (12 weeks) | Composite frailty score | Significant group-by-time improvement (p = 0.009, partial eta-squared = 0.073); instrument not a validated frailty scale; no confidence intervals reported | Open access; extraction verified |
| Bleijenberg et al. (2017), Netherlands | Three-arm cluster randomised trial | Community-dwelling older adults screened with an electronic frailty index (39 practices, n = 3,092 of 7,638 invited, 40.5%) | Nurse-led | Frailty screening plus nurse-led proactive care programme based on comprehensive geriatric assessment | Daily functioning, quality-adjusted life-years, costs | No frailty-status outcome; favourable cost-effectiveness probability for the nurse-led arm | Full text verified from published record (JAMDA 2017;18:1029-1036): single-blind, 3-armed cluster RCT, 39 practices, n = 3,092; frailty index >= 0.20; probability of cost-effectiveness at EUR 20,000/QALY 91% (nurse-led vs usual care), 55% (vs screening + GP care) |
| Bleijenberg et al. (2016), Netherlands | Mixed-methods fidelity study | Primary care older adults in the U-PROFIT programme (n = 835 in the nurse-led care arm) | Nurse-coordinated | Nurse-led proactive programme components | Fidelity of component delivery | Variable delivery of components; nutrition counselling least often delivered | Full text verified from published record (Worldviews Evid Based Nurs 2016;13(1):75-84): mixed-methods; 835 older persons; most nursing care delivered for falls risk and urinary incontinence; patients with nutrition problems seldom received nursing interventions |
| Muntinga et al. (2015), Netherlands | Fidelity and implementation study within a stepped-wedge cluster randomised trial | Frail community-dwelling older adults (composite geriatric screening; Geriatric Care Model) | Nurse-coordinated | Geriatric assessment and multidisciplinary care | Adherence to assessments and care plans | Assessment adherence initially high then declined; multidisciplinary consultation adherence improved over time | Open access; extraction verified |
| Ettema et al. (2015), Netherlands | Mixed-methods feasibility study | Older cardiac surgery candidates aged 65 years or older (prehabilitation); n = 70 of 114 eligible completed | Nurse-delivered | Prehabilitation (PREDOCS): exercise (breathing), nutrition, education; one nursing consult 2-4 weeks before surgery | Feasibility outcomes (MRC guidelines; CReDECI) | Feasible; integration into routine cardiac care difficult | Full text verified from author's doctoral thesis chapter and published record (Eur J Cardiovasc Nurs 2015;14(4):325-335): mixed-methods multicentre, 3 hospitals; nurses satisfied with tools; additional consult experienced as tiresome; cost-effective if 6-16 of 1,000 patients avoid complications |
| Azzellino et al. (2026a), Italy | Retrospective cohort | Frail older inpatients (Brass scale 11 or more), n = 200 | Nurse-coordinated | Integrated discharge pathway: early home care activation plus nurse case management | 30-day readmission | Nurse case manager involvement associated with lower readmission (odds ratio 0.023, 95% confidence interval 0.008 to 0.064, p < 0.001) | Open access; extraction verified |
| Ron et al. (2025), Israel | Prospective observational study with comparison group | Older adults with elevated frailty risk before surgery (validated screening) | Nurse-delivered | Comprehensive geriatric assessment plus prehabilitation delivered by a geriatric nurse | Activities of daily living and instrumental activities of daily living | Non-significant between-group differences (p = 0.13 to 0.15) | Open access; extraction verified |
| Cypel et al. (2026), Israel | Retrospective cohort | Adults aged 65 years or older at frailty risk (validated prediction model), n = 135,445 | Nurse-coordinated | Comprehensive geriatric assessment plus nursing follow-up programme | Hip fractures, hospitalisation days, vaccination coverage | Before-after improvements (hip fractures down 46.77%); no concurrent control group | Open access; extraction verified |

*MMAT = Mixed Methods Appraisal Tool; ADL = activities of daily living; GFI = Groningen Frailty Indicator.*

## **Section B. Primary studies newly identified in the updated PubMed search (20 September 2026) and integrated into the analytic corpus (n = 9)**

The following studies were identified by the updated PubMed search, retrieved as full texts and adjudicated as meeting all revised eligibility criteria (documented frailty or pre-frailty criterion; defined nursing involvement; multicomponent or comprehensive geriatric assessment-based intervention; 2015 onward; older adults). Following the final author decision of 20 September 2026, all nine studies are integrated into the analytic corpus, which totals 23 primary studies.

| **Study (country)** | **Design** | **Population and frailty criterion** | **Nursing role** | **Intervention components** | **Frailty-related outcome** | **Reported result** | **Verification** |
| --- | --- | --- | --- | --- | --- | --- | --- |
| Jepma et al. (2021), Netherlands | Randomised controlled trial (multicentre) | Hospitalised older adults, frailty screening (Discharge Screening for Community-dwelling frail older adults, >= 2 points), n = 306; mean age 82.4 | Nurse-coordinated | Care Chain Bailiwick: nurse-coordinated transitional care - comprehensive geriatric assessment, case management, home-based cardiac rehabilitation | ADL functioning, mortality, readmission (frailty not assessed as outcome) | Sustained ADL benefit at 12 months in the main trial report | Full text obtained and adjudicated; integrated into the analytic corpus (final author decision, 20 September 2026) |
| Safari et al. (2023), United Kingdom | Pilot randomised controlled trial (mixed-methods) | Community-dwelling older adults with confirmed frailty (assessed by practice nurses), n = 72 randomised (35/37) | Nurse-led (advanced nurse practitioner) | Advanced nurse practitioner-led tailored comprehensive geriatric assessment | Feasibility/acceptability; frailty and functional outcomes | Feasible and acceptable; frailty outcomes reported in pilot | Full text obtained and adjudicated; integrated into the analytic corpus (final author decision, 20 September 2026) |
| Hayes et al. (2025), Ireland | Prospective cohort | Community-dwelling older adults, Clinical Frailty Scale 4-6, n = 303 | Nurse-delivered (2 advanced nurse practitioners + 1 clinical nurse specialist per hub) | Community Support Team: interdisciplinary comprehensive geriatric assessment incl. nursing roles | Functional status; healthcare utilisation | Functional improvement reported in prospective cohort | Full text obtained and adjudicated; integrated into the analytic corpus (final author decision, 20 September 2026) |
| van Lieshout et al. (2018), Netherlands | Randomised controlled trial | Community-dwelling pre-frail or frail older adults (Groningen Frailty Indicator >= 1), n = 281 (139/142) | Nurse-delivered (partial; psychosocial component) | SPRY programme: medication review, physical fitness, social skills (community nurse), nutrition | Daily functioning (Katz-6); well-being | No between-group difference in daily functioning at 12 months | Full text obtained and adjudicated; integrated into the analytic corpus (final author decision, 20 September 2026) |
| Ha and Park (2020), Republic of Korea | Quasi-experimental (non-equivalent control) | Community-dwelling pre-frail older adults aged 65 years or older, n = 40 | Nurse-led | Person-centred nursing intervention: exercise, nutrition, mental health education (multicomponent) | Frailty (Korean frailty instrument); physical function | Frailty and physical function improved in intervention group | Full text obtained and adjudicated; integrated into the analytic corpus (final author decision, 20 September 2026) |
| Kasa et al. (2024), Ethiopia | Quasi-experimental (single group) | Community-dwelling frail older adults, n = 66 | Nurse-led | Six-session nurse-led programme: education across physical, nutritional, psychological and social domains | Frailty, depression, quality of life, activities of daily living | Frailty and ADL improved post-intervention | Full text obtained and adjudicated; integrated into the analytic corpus (final author decision, 20 September 2026) |
| Kang et al. (2025), Republic of Korea | Single-group pre-experiment | Community-dwelling older adults aged 65+, 100% pre-frail or frail (EHIS frailty instrument, mean 8.32 +/- 2.85), n = 92 | Nurse-led (community health nurse) | Multi-faceted group programme: education + exercise + social participation | Frailty (EHIS); self-efficacy | Frailty score improved significantly post-programme | Full text obtained and adjudicated; integrated into the analytic corpus (final author decision, 20 September 2026) |
| Lee et al. (2025), Republic of Korea | Quasi-experimental (pragmatic, mixed-methods) | Community-dwelling pre-frail older adults (Korean frailty scale), n = 40 | Nurse-led | Nursing Frailty Prevention programme: nurse counselling + group education + exercise + walking | Frailty; physical and psychosocial measures | Frailty and physical measures improved; qualitative themes on programme value | Full text obtained and adjudicated; integrated into the analytic corpus (final author decision, 20 September 2026) |
| Yoo et al. (2026), Republic of Korea | Single-arm pilot (pre-post) | Nursing home residents aged 65+ (Fried phenotype; Korean Mini-Mental State Examination >= 18), n = 27 | Nurse-led | Nurse-led peer support programme: motor-cognitive activities + peer support (6 weeks) | Feasibility; handgrip strength; depressive symptoms; social frailty | Feasible and acceptable; within-group changes in social frailty and secondary outcomes | Publisher record verified via DOI (Springer); integrated into the analytic corpus (final author decision, 20 September 2026) |

A further 17 reports judged potentially eligible at abstract level could not be retrieved as full texts (paywalled despite retrieval attempts via open-access repositories, author manuscripts and DOI resolution) and are documented in Supplementary Table S4; six additional evidence syntheses identified in the updated search await full-text assessment.
