# Supplementary material for: Multicomponent nursing interventions and frailty-related outcomes in older adults: An integrative review of primary studies and evidence syntheses
Source: Int J Nurs Stud Adv. 2026 Oct 1;11:100687. doi: 10.1016/j.ijnsa.2026.100687 (PMC13675801; doi:10.1016/j.ijnsa.2026.100687)
Supplement: Supplementary file 8 [file mmc8.docx]

**Supplementary Table S2**

*Methodological quality appraisal of the primary studies using the Mixed Methods Appraisal Tool (MMAT)*

Manuscript IJNSA-D-26-00510R1. Multicomponent Nursing Interventions and Frailty-Related Outcomes in Older Adults: An Integrative Review of Primary Studies and Evidence Syntheses.

Ratings follow the MMAT (2018) design-specific criteria; no composite scores were computed and no studies were excluded on the basis of methodological quality. Part 1 summarises the appraisal findings reported in the manuscript (Section 3.2). Part 2 records design-specific ratings per study. Y = yes; CT = can't tell; N = no; design-specific screening questions (S1, S2) were satisfied for all included studies.

## **Part 1. Appraisal summary by design**

| **Design (n)** | **MMAT criteria** | **Appraisal finding** |
| --- | --- | --- |
| Randomised controlled trials (n = 9) | Randomisation process | Appropriate in all nine trials |
|  | Allocation concealment | Could not be confirmed from the published reports of three trials (can't tell) |
|  | Blinding of outcome assessors | Documented in three trials |
|  | Completeness of outcome data | Acceptable in all nine trials |
| Quasi-experimental (n = 7) | Confounding | Addressed analytically in two (mixed-model adjustment in Song and Boo 2022; propensity-score matching in Zhou et al. 2025); neither achieved randomised comparability, and the groups in Zhou et al. (2025) were imbalanced at baseline on frailty status. The five studies added in the updated search (Ha and Park 2020; Kasa et al. 2024; Kang et al. 2025; Lee et al. 2025; Yoo et al. 2026) used non-equivalent control or single-group designs, three without a control group |
| Mixed-methods (n = 3) | Integration of quantitative and qualitative components | Appropriate in all three |
| Observational (n = 4) | Confounding | Addressed statistically in Azzellino et al. (2026a); not addressed in Cypel et al. (2026) (before-after design without concurrent control group); Ron et al. (2025) used a non-randomised comparison group and a small sample; Hayes et al. (2025) was a single-arm prospective cohort without a concurrent control group |

## **Part 2. Study-level appraisal record**

| **Study** | **Design (MMAT category)** | **Key appraisal notes** |
| --- | --- | --- |
| Faria et al. (2023) | RCT | Randomisation appropriate; completeness acceptable |
| Yas and Secginli (2025) | RCT | Randomisation appropriate; completeness acceptable |
| Kim et al. (2025) | RCT (pilot) | Randomisation appropriate; pilot trial, completeness acceptable |
| Luo et al. (2024) | RCT | Randomisation appropriate; completeness acceptable |
| Huang et al. (2025) | RCT | Randomisation appropriate; outcome assessor blinding documented; completeness acceptable |
| Bleijenberg et al. (2017) | Cluster RCT | Single-blind, cluster-randomised; allocation by practice; assessor blinding documented; completeness acceptable (1-year follow-up) |
| Zhou et al. (2025) | Quasi-experimental | Propensity-score matching; baseline frailty imbalance between groups retained |
| Song and Boo (2022) | Quasi-experimental | Mixed-model adjustment for confounding; non-equivalent control group |
| Bleijenberg et al. (2016) | Mixed-methods | Quantitative fidelity data collected during trial plus post-trial focus group; integration appropriate |
| Muntinga et al. (2015) | Mixed-methods | Fidelity measurement embedded in stepped-wedge trial; integration appropriate |
| Ettema et al. (2015) | Mixed-methods | MRC feasibility framework; patient satisfaction telephone interviews plus nurse/manager interviews; integration appropriate |
| Azzellino et al. (2026a) | Observational (retrospective cohort) | Multivariable adjustment for readmission outcome |
| Ron et al. (2025) | Observational (prospective, comparison group) | Non-randomised comparison; small sample |
| Cypel et al. (2026) | Observational (before-after) | No concurrent control group; secular trends cannot be excluded |
| Jepma et al. (2021) | RCT | Randomisation appropriate; completeness acceptable; nurse-coordinated transitional care programme |
| van Lieshout et al. (2018) | RCT | Randomisation appropriate; completeness acceptable |
| Safari et al. (2023) | RCT (pilot, mixed-methods) | Randomisation appropriate in the pilot trial; integration of quantitative and qualitative components appropriate |
| Ha and Park (2020) | Quasi-experimental | Non-equivalent control group; no statistical adjustment for confounding |
| Kasa et al. (2024) | Quasi-experimental | Single-group design without a control group |
| Kang et al. (2025) | Quasi-experimental | Single-group pretest-posttest design without a control group |
| Lee et al. (2025) | Quasi-experimental | Pragmatic trial; non-equivalent groups; allocation procedures pragmatic |
| Yoo et al. (2026) | Quasi-experimental | Single-arm pilot pre-post design without a control group |
| Hayes et al. (2025) | Observational (prospective cohort) | Single-arm prospective cohort; no concurrent control group; covariate adjustment for outcome prediction |

*Note. Per-criterion responses for allocation concealment and assessor blinding in individual trials, and full MMAT checklists, are held in the authors' appraisal records and are available on request. RCT = randomised controlled trial.*
